# Health benefits of decreases in on-road transportation emissions in the United States from 2008 to 2017

**DOI:** 10.1073/pnas.2107402118

**Published:** 2021-12-13

**Authors:** Ernani F. Choma, John S. Evans, José A. Gómez-Ibáñez, Qian Di, Joel D. Schwartz, James K. Hammitt, John D. Spengler

**Affiliations:** ^a^Population Health Sciences, Harvard University, Boston, MA 02115;; ^b^Department of Environmental Health, Harvard T.H. Chan School of Public Health, Boston, MA 02115;; ^c^Harvard Kennedy School of Government, Cambridge, MA 02138;; ^d^Vanke School of Public Health, Tsinghua University, Beijing 100084, China;; ^e^Department of Health Policy and Management, Harvard T.H. Chan School of Public Health, Boston, MA 02115;; ^f^Toulouse School of Economics, Université Toulouse Capitole, 31080 Toulouse, France

**Keywords:** particulate matter, transportation, air pollution, public health, climate change

## Abstract

Decades of air pollution regulation have yielded enormous benefits in the United States, but vehicle emissions remain a climate and public health issue. Studies have quantified the vehicle-related fine particulate matter (PM_2.5_)-attributable mortality but lack the combination of proper counterfactual scenarios, latest epidemiological evidence, and detailed spatial resolution; all needed to assess the benefits of recent emission reductions. We use this combination to assess PM_2.5_-attributable health benefits and also assess the climate benefits of on-road emission reductions between 2008 and 2017. We estimate total benefits of $270 (190 to 480) billion in 2017. Vehicle-related PM_2.5_-attributable deaths decreased from 27,700 in 2008 to 19,800 in 2017; however, had per-mile emission factors remained at 2008 levels, 48,200 deaths would have occurred in 2017. The 74% increase from 27,700 to 48,200 PM_2.5_-attributable deaths with the same emission factors is due to lower baseline PM_2.5_ concentrations (+26%), more vehicle miles and fleet composition changes (+22%), higher baseline mortality (+13%), and interactions among these (+12%). Climate benefits were small (3 to 19% of the total). The percent reductions in emissions and PM_2.5_-attributable deaths were similar despite an opportunity to achieve disproportionately large health benefits by reducing high-impact emissions of passenger light-duty vehicles in urban areas. Increasingly large vehicles and an aging population, increasing mortality, suggest large health benefits in urban areas require more stringent policies. Local policies can be effective because high-impact primary PM_2.5_ and NH_3_ emissions disperse little outside metropolitan areas. Complementary national-level policies for NO_x_ are merited because of its substantial impacts—with little spatial variability—and dispersion across states and metropolitan areas.

## Significance

Despite decades of reductions in vehicle emissions in the United States, their impacts remain large, and the offsetting effects of different factors on benefits achieved in recent years are not well understood. We assess benefits from 2008 to 2017 on a fine spatial resolution using the latest epidemiological evidence and emissions inventories. We find that regulation continues to yield large benefits: $270 (190 to 480) billion in 2017 from reduced PM_2.5_-attributable mortality and greenhouse gas emissions. Traffic-related PM_2.5_-attributable mortality would have been 2.4 times as large in 2017 if vehicles had still been emitting at 2008 levels, accounting for most benefits. Urban passenger light-duty vehicles have become increasingly important, and major health gains require more stringent policies to curb their emissions.

Health impacts of air pollution from transportation remain a major public health problem in the United States with several studies estimating roughly 17,000 to 20,000 deaths/year attributable to it in recent years, the vast majority from fine particulate matter (PM_2.5_) (1–4). Researchers have used different methods to estimate this burden, limiting comparability among estimates, but those who have estimated attributable deaths in different years have shown this burden has decreased. Dedoussi et al. (3) estimate that they were cut in half in the 2005 to 2018 period, from 37,000 to 18,400 due to PM_2.5_ and ozone, whereas Fann et al. (1) estimate just under 30,000 in 2005 and 19,300 in 2016, a decrease of about a third. These studies’ estimates for 2016 and 2018, however, rely on forecasts of emissions made years in advance.

Transportation emissions also contribute to climate impacts. Transportation greenhouse gas (GHG) emissions have increased in recent years, and they were responsible for 28% of the US GHG emissions in 2018 (5). A total of 83% of transportation GHG emissions in 2018 came from vehicles, and 70% of vehicle GHG emissions came from light-duty vehicles (LDVs) (5). In recent years, LDV energy efficiency has increased and GHG emission factors per mile (EF) decreased, but their overall climate impacts have increased (5, 6). Increased market penetration of larger LDVs (6) and increased vehicle miles traveled (VMT) (7) have contributed to this overall increase.

Decades of environmental regulation in the United States have drastically reduced emissions from vehicles by as much as 99% per vehicle for common pollutants since 1970 (8). Transportation emissions are one element of a substantial effort to reduce ambient PM_2.5_ in recent decades (9, 10), following regulation of air pollution that has been cost-beneficial and has yielded substantial benefits. The US Environmental Protection Agency (EPA) (11) estimates that the Clean Air Act Amendments of 1990 have yielded $2 trillion/year (2006 US dollars) in benefits from all sectors in 2020, or 30 times its cost, with 90% of the benefits coming from reduced PM_2.5_-attributable mortality. Fuel efficacy standards and vehicle emission controls have been responsible for a substantial part of these benefits.

Benefits of recent reductions in vehicle emissions, on the other hand, are not well understood. Several studies have quantified mortality from on-road transportation in recent years (1–4, 12–15), some of them also assessing changes over time and showing decreases. To our knowledge, however, no study has carried out a fine-scale assessment relying on counterfactual scenarios that capture changes in fleet composition and VMT, population, age-specific baseline mortality rates, and lower ambient PM_2.5_ concentrations at baseline. The latter is important because more recent epidemiological evidence from the Global Exposure Mortality Model (GEMM) (16) suggests a nonlinear function linking ambient PM_2.5_ concentration to mortality. The GEMM concentration–response function (CRF) is concave, exhibiting higher marginal effects at lower concentrations. As ambient PM_2.5_ concentrations in the United States have dropped in recent decades (10), this nonlinearity suggests marginal effects are increasing over time. The previously widely used Global Burden of Disease (GBD) Integrated Exposure-Response (IER) model (17, 18) also estimated a concave CRF, but GEMM estimates more than twice as many attributable deaths for the United States and Canada when compared to GBD IER. GEMM also includes more recent evidence from epidemiological studies of populations in the two countries that allow it to estimate mortality risks for exposures to very low ambient PM_2.5_ concentrations—as low as 2.4 μg/m^3^, lower than previous models—that are relevant for policies in the United States.

Vehicle impacts also exhibit large spatial variability across states and cities (19, 20). Metropolitan areas are especially important because previous research has suggested that impacts per mile of passenger vehicles driving in these areas are large (20), and passenger transportation is now responsible for more PM_2.5_-attributable deaths in the United States than truck use (4). Spatial variability in impact suggests a potential for more stringent policies in metropolitan areas where impacts are higher, but considering local policies would require understanding local impacts versus those transported to and affecting populations in other areas. Previous research has shown that over a third of impacts caused by all vehicle emissions in the United States occur across state lines, mostly from NO_x_ emissions (3); nevertheless, transfers of impacts caused by vehicles in metropolitan areas are not well studied.

This paper assesses benefits of recent emissions reductions of on-road transportation in the contiguous United States occurring between 2008 and 2017. We assess impacts on a fine scale using a nonlinear CRF from the most recent epidemiological evidence from GEMM (16). We combine 1-km–resolution baseline ambient PM_2.5_ levels (21), fine-scale (1 km in densely populated areas) air pollution modeling (2, 22), and county-level age- and cause-specific mortality (23). We assess impacts in 2017 for four counterfactual emission scenarios (2008 EFs, 2011 EFs, 2014 EFs, and 2017 EFs), each using county-level EFs for each pollutant and 13 vehicle types from the respective year’s National Emissions Inventory (NEI) (24–27). Our combination of fine-scale modeling and counterfactual emission scenarios allows us to capture changes in demographics, fleet composition, and baseline ambient PM_2.5_ levels. We estimate benefits from decreases in PM_2.5_-attributable mortality due to reductions in on-road transportation emissions of primary PM_2.5_, SO_2_, NO_x_, NH_3_, and volatile organic compounds (VOCs) (air pollution) and climate benefits from reductions in on-road transportation emissions of CO_2_, CH_4_, and N_2_O (GHGs). As passenger vehicles were previously estimated to be responsible for most of the burden, we present a spatially explicit analysis of passenger LDVs with a focus on 53 large metropolitan statistical areas (MSAs), which we define as those with population exceeding 1 million in 2017 according to the US Census Bureau (28). In 2017, these 53 MSAs accounted for 56% of the US population (29) and 50% of the US VMT from all road vehicles (27). We refer to these large MSAs simply as MSAs or metropolitan areas throughout the paper.

## Results

### Emission Changes.

**Fig. 1.**
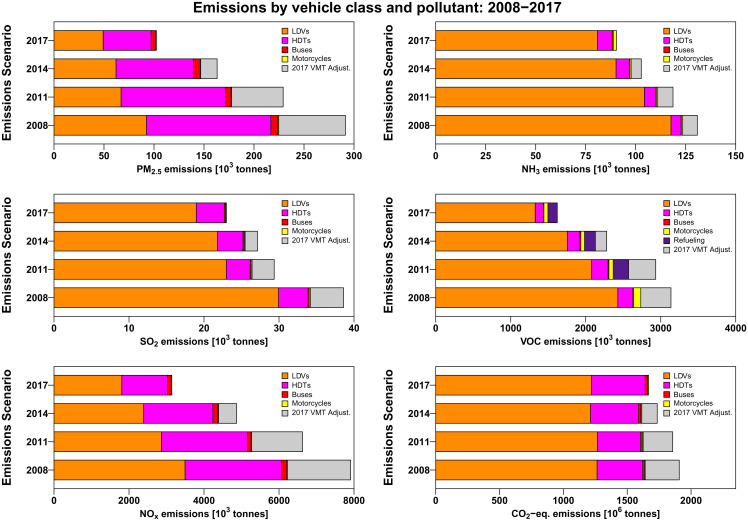
Emissions in the 2008 to 2017 period by pollutant and vehicle category. Unlike the three most recent NEIs, the 2008 NEI (24) does not present refueling emissions separately. The color bars represent actual emissions in each year, whereas the light gray represents the amount added when VMT is adjusted to 2017 levels. 1 tonne = 1 metric ton.

Fig. 1 shows emissions of GHGs and each air pollutant by vehicle class in the four emission scenarios as well as the effect of the adjustment of VMT to 2017 levels. Emissions for all air pollutants decreased substantially each year. In contrast, GHG emissions increased during the 2008 to 2017 period, although very modest reductions would have been observed if VMT had remained constant at 2008 levels. Primary PM_2.5_ and NO_x_ experienced the largest reductions—emissions using 2017 EFs were only 35% and 40% as large as they would have been using 2008 EFs. Heavy-duty truck (HDT) emissions were the main source of reductions, with smaller declines observed for the pollutants for which HDTs are relatively unimportant sources. Increases in VMT during the period added between 6% (NH_3_) and 30% (primary PM_2.5_) to 2008 emissions. In comparison, total fleet VMT increased by just 7% on a national level (*SI Appendix*, Section 2), but most of the effect on emissions is due to changes in fleet composition, including fewer car VMT and more passenger truck and HDT VMT.

### Health and GHG Impacts.

**Fig. 2.**
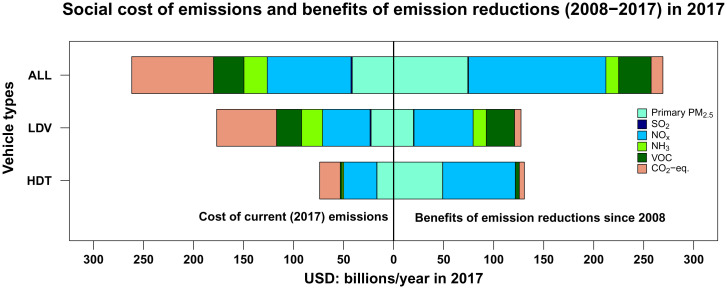
Social cost of emissions in 2017 (*Left*) and benefits achieved since 2008 (*Right*) for GHGs and each air pollutant. If vehicles were emitting per mile as they were in 2008, benefits would not have occurred and impacts in 2017 would have been represented by the full bars (i.e., benefits shown on right side of the graph represent avoided costs; had those costs occurred, they would have been added to social costs of emissions in 2017).

We estimate that in 2017, the social cost of on-road emissions—the sum of monetary damages of mortality attributable to PM_2.5_ and climate change damages—was $260 billion; however, if vehicles were still emitting at 2008 levels (per mile), this cost would have been $530 billion. Decreases in EFs since 2008 are therefore responsible for benefits of $270 billion per year in 2017, 95% of which is from air pollutants (Fig. 2). We estimate 19,800 deaths attributable to PM_2.5_ from transportation emissions in 2017, which account for 69% of the current $260 billion impact. This figure would have been 2.4 times as high (48,200 or $440 billion) under 2008 EFs. For each air pollutant species, the percentage decreases in mortality are similar to decreases in emissions. NO_x_ emissions are responsible for a majority (53%) of air pollution benefits, but, despite drastic reductions, the pollutant still contributed 46% of impacts in 2017. Unlike air pollution, progress on reducing GHG impacts has been slow; hence, their share of overall impacts has increased, and they are responsible for a third of overall impacts in 2017.

**Fig. 3.**
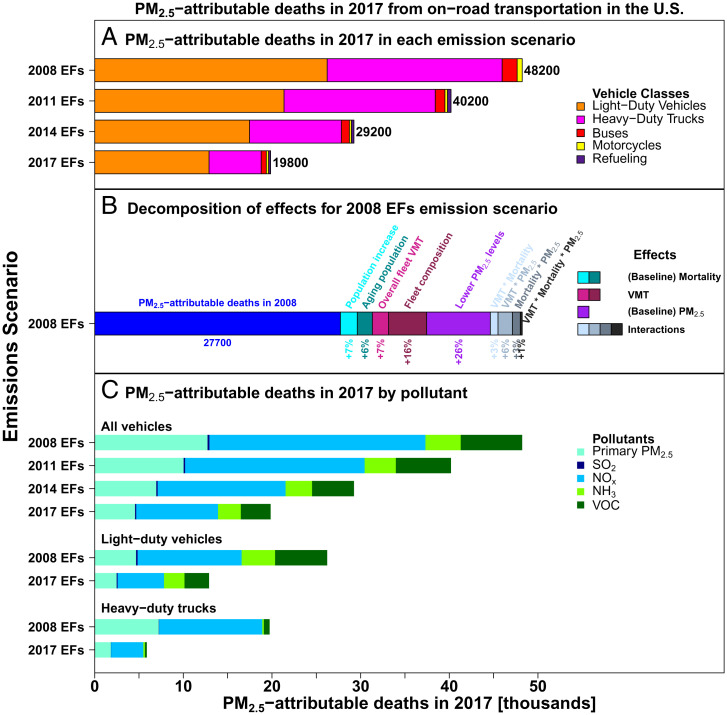
PM_2.5_-attributable deaths caused by vehicle emissions in 2017, in each of the four vehicle emissions scenarios. *A* shows the impacts by vehicle class. *B* shows the decomposition of effects over the 2008 to 2017 period, for the 2008 EFs scenario. *C* shows the impacts for each pollutant for the entire fleet as well as separately for LDVs and HDTs.

Fig. 3 shows PM_2.5_-attributable deaths from vehicle emissions in 2017 under the four emission scenarios considered (2008 EFs, 2011 EFs, 2014 EFs, and 2017 EFs) and the portion due to each vehicle class and air pollutant species. We estimate that if vehicles were still emitting at 2008 levels, they would have been responsible for 48,200 deaths in 2017, 2.4 times as many as the actual 19,800 that occurred after improvements. Fig. 3 also shows the importance of capturing the mortality effects of changes in baseline ambient PM_2.5_ levels, VMT, and increased baseline mortality. We estimate that vehicles emitting at 2008 levels only caused 27,700 PM_2.5_-attributable deaths in 2008, but those would have been 74% larger in 2017 had emission factors not decreased. Benefits of emissions reductions would have been greatly underestimated if the simple difference in attributable deaths between the years had been used without accounting for proper counterfactual scenarios.

The 74% increase in impacts from 2008 to 2017 using the same 2008 emission factors is explained by lower ambient PM_2.5_ levels in 2017, causing a 26% increase, followed by VMT effects, which include increases in overall VMT and changes in fleet composition (22%), and increased baseline mortality (13%). Interactions among the effects add the remaining 13%. Individual effects of PM_2.5_, VMT, and mortality reflect changes in impact in the 2008 EFs scenario when each of these factors is changed to 2017 levels and the others are held constant at 2008 levels. Two-way interactions reflect changes in impact when two factors are changed minus the sum of their individual effects, and the three-way interaction reflects the total effect minus the effects of individual components and two-way interactions. All interactions are positive since individual effects are positive and, when two or more occur, there is a compound effect.

Lower baseline PM_2.5_ levels result in higher slopes (i.e., percentage increase in mortality per an increase in ambient level of 1 μg/m^3^) from GEMM’s CRF, which is nonlinear and concave in PM_2.5_ concentrations. Higher slopes consequently yield higher marginal impacts per mass emitted. We estimate that US population-weighted levels dropped from 10.5 μg/m^3^ in 2008 to 7.5 μg/m^3^ in 2017, similar to the decrease from 10.9 to 7.7 μg/m^3^ that the EPA estimates over the period (10). Our model uses 1-km–resolution baseline ambient PM_2.5_ concentrations and age-specific GEMM hazard ratios; therefore, the increase in impact is not uniform across the country. Overall, lower baseline PM_2.5_ levels yielded a 26% increase in overall impacts in the 2008 EFs scenario when baseline mortality and VMT remained constant, which is similar to the increase in the slope of GEMM at the mean population exposures. If we take the GEMM version that applies to all adult nonaccidental mortality, the slope increases from 0.84% at 10.5 μg/m^3^ to 1.05% at 7.5 μg/m^3^, or 26%.

Increases in baseline mortality cause an approximately proportional increase in the impacts per mass emitted (see *Materials and Methods*, Eqs. **1** and **2**). Adult nonaccidental mortality, to which GEMM’s CRF applies, rose 14%, from 2.2 million in 2008 to 2.5 million in 2017 (23). This yielded a 13% increase in impacts in the 2008 EFs scenario when VMT and baseline PM_2.5_ concentrations remained unchanged from 2008 levels. We explored how much of this effect was due to population growth and how much was due to changes in mortality rates. For population, we use the growth in total population in the contiguous United States, which grew by 6.9%, from 302 million in 2008 to 323 million in 2017, causing a proportional increase of 6.9% in impacts. Assessing impacts using county-specific population growth yields the same 6.9% increase in impacts. The remainder of the effects are due to higher adult nonaccidental mortality rates, which grew by 6.4%, from 733 to 780 per 100,000 population. The increase in mortality rates is due to an aging population. Age-specific mortality rates declined for all GEMM age groups other than 30 to 34 y old, with a median decrease of 7% in the other age groups, but the change in the age structure of the population was substantial. While the population younger than 60 y grew by just 2%, the population between 60 and 75 y grew by 38%, and the population over 75 y grew by 16%.

VMT effects increase impacts through two components: increases in overall miles traveled of all vehicle types and a shift in the fleet composition to heavier, more polluting vehicles. When impacts for the 2008 EFs scenario are estimated with 2017 VMT for each county and vehicle type, impacts are 22% higher than with 2008 VMT values, holding both baseline mortality and ambient PM_2.5_ concentrations constant at 2008 levels. Nationwide increases in overall VMT account for only about a third of effects, as VMT grew by 6.6%, from 2.97 trillion miles in 2008 to 3.16 trillion miles in 2017, and the impacts increase proportionally with it. The remaining 70% is due to changes in fleet composition, which increased impacts by 15.5%. This is due to an increase of 30% in the VMT of light trucks and heavy-duty vehicles, whereas car VMT decreased by 15%. Overall, VMT changes from all vehicle types on a county level cause an effect (+5.5%) similar to that of national-level changes (+6.6%), further supporting the claim that the change in fleet composition is the most important component and not the change in the spatial pattern of travel. Finally, there has been substantial change in the VMT of light commercial trucks between NEIs 2011 and 2014 that make it difficult to apportion effects between LDVs and HDTs, but this likely does not affect the total effects of fleet composition changes substantially (*SI Appendix*, Section 2).

The reduction in HDT emissions in the 2008 to 2017 period was substantial and deserves emphasis. The slower progress in curbing LDV impacts has made them responsible for a majority (65%) of transportation air pollution impacts in 2017, 93% of which are from passenger LDVs. *SI Appendix*, Fig. S1 shows that in 2017, PM_2.5_-attributable deaths from passenger LDVs under 2008 EFs (24,100) would have been twice as large as what they were with 2017 EFs (12,000) (*SI Appendix*, Fig. S1). In comparison, HDT impacts in 2017 under 2008 EFs would have been 3.4 times as large as they were with 2017 EFs (Fig. 3). While reductions in passenger LDV emission factors were substantial and avoided this doubling of impacts, attributable deaths decreased by just 16%, from 14,300 in 2008 (with 2008 EFs) to 12,000 in 2017 (with 2017 EFs). This is a consequence of the effects of lower PM_2.5_ concentrations, higher baseline mortality, and VMT, which would have caused an increase of 68% in the impacts from 2008 to 2017 if vehicles were still emitting at 2008 levels (2008 EFs).

**Fig. 4.**
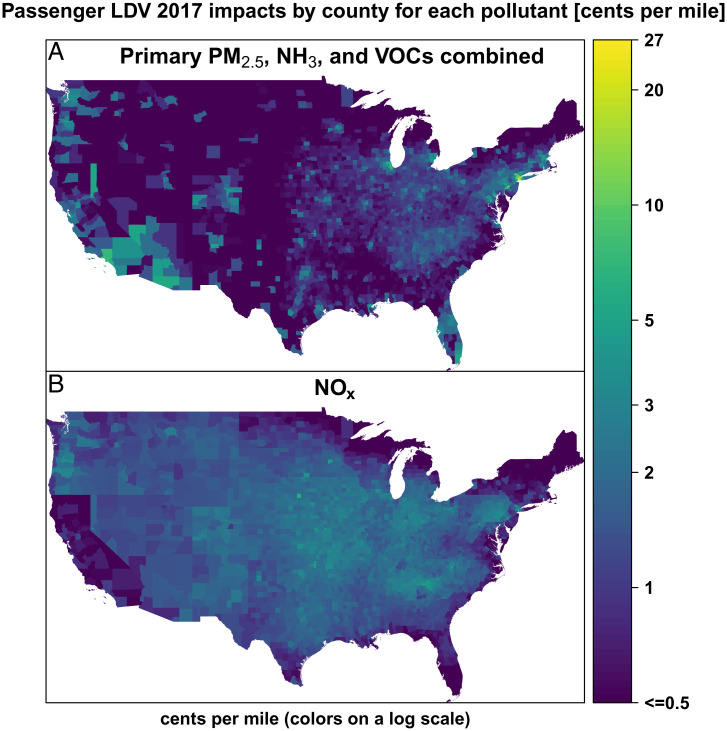
Impacts per mile by county for passenger LDVs. SO_2_ impacts are not shown since it represents just 0.9% of their overall impacts. *A* shows the combined impacts of emissions of primary PM_2.5_, NH_3_, and VOCs. *B* shows the impact of NO_x_ emissions. Although impacts per mile can be as low as 0.01 cent/mile, no differentiation for values smaller than 0.5 is shown. US County Boundaries from US Census Bureau (30).

Health impacts per mile of passenger LDV emissions exhibit substantial spatial variability (Fig. 4 and *SI Appendix*, Fig. S2). In 2017, passenger LDVs driven in metropolitan areas accounted for 51% of the passenger LDV VMT and 64% of their impacts, exceeding the impacts of all the HDTs in the United States combined by more than 30%. Passenger LDVs driving in large MSAs cause higher health impacts per mile (5.1 cents) than those driving outside them (3.0 cents) (*SI Appendix*, Table S4). This difference of 2.1 cents/mile is predominantly due to primary PM_2.5_ and NH_3_ emissions, whose impacts are higher in large MSAs by 0.9 and 0.8 cents/mile, respectively, accounting for 43% and 38% of the difference. NO_x_ impacts per mile, on the other hand, are very similar within and outside large MSAs, even as the pollutant is responsible for 40% of all the PM_2.5_ burden of passenger LDVs. Finally, GHG impacts are substantial but show little spatial variability and are the same within and outside large MSAs, since climate change impacts per mass emitted are independent of location and there is little variability in EFs.

A portion of health impacts occur in regions other than the region where the emissions occurred, especially in the case of secondary particles, since SO_2_, NO_x_, NH_3_, and VOCs are emitted as gases and form particles in the atmosphere. Dedoussi et al. (3) estimated that 38% of the impacts from PM_2.5_ and ozone from on-road transportation emissions occurred across state lines (i.e., in a state different from where the emissions took place). Their estimate was based on an analysis with a relatively course spatial resolution of 55 km, which limited their ability to estimate transfers of impacts across relatively small geographies such as metropolitan areas. With our fine spatial resolution, we show that transfers of impacts from passenger LDVs in metropolitan areas are very limited: 83% of the impacts from passenger LDV emissions in large MSAs occur within the same MSA (*SI Appendix*, Fig. S3), including 66% for NO_x_ emissions and 91% for the other air pollutants combined. The share of health impacts from passenger LDVs driven in large MSAs that occurs in other regions (17%) is smaller than the share of all passenger LDV impacts that occurs across state lines (23%). This suggests that some of the cross-state pollution highlighted by Dedoussi et al. (3) remains within the same MSA because many MSAs span multiple states. We estimate fewer transfers of impacts across state lines (24% for all on-road transportation) than Dedoussi et al. (3), in part because we do not include ozone; however, it could also reflect our ability to better estimate transfers among small states.

## Discussion

### Uncertainty in Health and Climate Impacts.

Uncertainties in our estimates come primarily from the uncertainty about the relationship linking ambient PM_2.5_ concentrations to mortality (the CRF) and about the social cost of carbon (SCC). Uncertainty about the value per statistical life (VSL) is also an important contributor to our monetized estimates of PM_2.5_-attributable mortality. To explore the impact of the uncertainty about the SCC, we compared our base results, which use the average SCC ($49/metric ton; see *Materials and Methods*), to alternative results using the high-impact scenario SCC ($144/metric ton), both values for 2020 emissions—with a 3% per year discount rate—from the Interagency Working Group on Social Cost of Greenhouse Gases (31). To explore the sensitivity to alternative interpretations of the evidence on the mortality impacts of PM_2.5_ exposure, we compared our base results, which use GEMM, to two alternative CRFs: the metaregression by Vodonos et al. (32, 33), which estimates a nonlinear CRF that yields higher effects, and the extended follow-up analysis of the American Cancer Society study by Krewski et al. (34), a large cohort in the United States often used in the literature, which yields lower effects. We implemented Krewski et al. (34) as a log-linear CRF (i.e., assuming the percent change in mortality per each μg/m^3^ is the same and valid for all ambient PM_2.5_ levels). We note that all three CRFs assume equitoxicity per mass emitted so that possible differential toxicity is not captured. The evidence for differential toxicity is not conclusive, but Vodonos et al. (32) find that particles from traffic are likely more toxic, on a mass basis, than the ambient mix. We also do not include the parametric uncertainty in these CRFs because it is much smaller than the uncertainty due to differences among the models.

Under the three CRFs and two SCCs, benefits range from $190 billion to $480 billion/year and current (2017) impacts from $210 to $550 billion/year (*SI Appendix*, Figs. S4 and S5). The three CRFs result in essentially a scaling of impacts, with shares for individual pollutants and vehicle classes remaining approximately constant. GEMM impacts are close to a geometric mean of the other two: ∼1.5 times as the value of Krewski et al. (34) and 1/1.7 times the value of Vodonos et al. (32) (*SI Appendix*, Fig. S6). The high-impact SCC also simply increases GHG impacts by roughly a factor of three. We do not apply an uncertainty range for the VSL, but a different VSL would simply result in a scaling of monetized air pollution impacts. The US Department of Health and Human Services (HHS) (35) recommends sensitivity analyses using a range of values within roughly ±50% of the mean.

Our central estimate of 19,800 deaths attributable to traffic-related PM_2.5_ emissions in 2017 is quite close to Fann et al.’s (1) estimate of 17,000 for 2016 and Dedoussi et al.’s (3) estimate of 18,400 (including ozone) for 2018. However, both studies used the CRF from Krewski et al. (34) and relied on emissions projected years in advance. Employing the CRF from Krewski et al. (34), our estimate of attributable deaths from traffic-related PM_2.5_ in 2017 would have been substantially smaller (13,600).

Other sources of uncertainty include those about the source–receptor matrix of the InMAP model (ISRM) and NEI emissions, but these have a smaller impact in our estimates. Goodkind et al. (2) report a mean fractional bias of −6% and a mean fractional error of 36% in predicting annual average PM_2.5_ at EPA monitoring locations in 2011, which includes all the uncertainty about the ISRM, its meteorological inputs, and NEI emissions. This uncertainty is substantially smaller than the factor of ∼2.5 difference in impacts under the different CRFs. InMAP was trained for 2005, so the prediction error is not constant over time, but the variation in error across years is small. InMAP’s error also varies spatially, but fortunately, for primary PM_2.5_, the main driver of spatial variability in impacts in our estimates, InMAP’s error is very small (2, 36). However, InMAP’s error for NH_3_ is larger and could add uncertainty to our estimates of spatial variability since NH_3_ is an important contributor to it.

### Policy Implications.

Although regulation of emissions continues to yield enormous benefits, our results indicate that to achieve further public health and climate gains, even more stringent policies will be required. While we have not conducted a benefit–cost analysis, benefits since 2008 ($190 to $480 billion/year) are an order of magnitude larger than all 1990 Clean Air Act Amendments compliance costs for on-road vehicles and fuels ($28 billion 2006 US dollars/year in 2020) (11). While these costs include all regulation, not only those that reduced EFs after 2008, the EPA’s report is already a decade old and might not include all recent rules that contribute to our benefit estimates.

If the health and climate impacts of transportation are to be reduced, passenger LDVs seem to be an attractive target. They cause a majority of both the public health and the climate burden. On their climate burden, progress was very small in reducing emission factors, and overall emissions have increased. With respect to air pollutants, progress in reducing their emissions was substantial—even if lower than for HDTs—but almost entirely offset by a combination of effects that have increased impacts per mass emitted: a higher market penetration of larger and more polluting passenger vehicles; new epidemiological findings, which suggest that the CRF for PM_2.5_-induced mortality may be concave, exhibiting larger marginal impacts at lower concentrations; and higher mortality rates at baseline as the population is aging. The consequence is that the health impacts of passenger LDVs in 2017 were just 16% smaller than they were in 2008, and drastic cuts in the future seem unlikely in the absence of more stringent policies. The market penetration of larger passenger vehicles has increased in recent years (6), and there are indications that marginal impacts of emissions may continue to increase because of growing population density and elevated mortality rates experienced by an aging population. Large MSAs—where most of the passenger LDV impacts accrue—saw a 31% increase in the population over 60 y old from 2008 to 2017. This is likely to have future effects on air pollution mortality as the population continues to age because this population increase is concentrated in the 60- to 69-year-old age groups, but most deaths attributable to air pollution occur in the population over 70 years old. On the other hand, ever-increasing marginal social costs per mass emitted, especially in large MSAs, make it more attractive to curb emissions and more likely that such efforts will be cost-beneficial.

The health impacts of passenger LDV emissions occur disproportionately in large metropolitan areas, and the combination of large spatial variability and local nature of these impacts in urban areas support local strategies to curb emissions. Passenger LDVs driving in urban areas cause high-impact emissions: health impacts per mile of passenger LDV driving in large MSAs are 73% larger than those driving outside them and account for 64% of the total passenger LDV burden. However, recent reductions in PM_2.5_-attributable mortality have been similar to overall emission cuts for each species, indicating that recent efforts have not taken advantage of the potential benefits of strategies that emphasize reductions of high-impact emissions in urban areas. As impacts per mass emitted in urban areas continue to increase, a focus on local strategies in these areas is merited. Strategies could include vehicle electrification (20) or other solutions aimed at reducing VMT or passenger vehicle use such as carpooling and investments in bicycle use mass transit ridership. National-level policies, however, have been successful in reducing emissions and remain important. In particular, national-level policies for NO_x_ are crucial because the pollutant remains a main source of impacts, but, unlike the other pollutants, it contributes little to spatial variability, and a substantial portion of NO_x_ impacts is exported across state and metropolitan area lines.

Efforts to reduce vehicle emissions and VMT will continue to have public health benefits, especially if cuts in emissions occur in metropolitan areas. Understanding which regulations or control strategies would be most beneficial, however, requires thorough analysis, including evaluation of costs and comparing them to benefits, which is beyond the scope of this paper.

## Materials and Methods

### Emissions.

We create four emission scenarios for emissions in 2017: actual emissions (2017 EFs) and three counterfactual scenarios in which county-level EFs for each vehicle type are the same as they were in 2014 (2014 EFs scenario), 2011 (2011 EFs), and 2008 (2008 EFs). Each scenario uses data for the NEI for the respective year (24–27), which is provided as total emissions (except refueling) and VMT by vehicle type, for each county. We calculate county-level EFs for each of the 13 vehicle types currently used by the EPA and apply them to their VMT in each county in 2017 (*SI Appendix*, Sections 1 and 2). These counterfactual scenarios therefore capture changes in fleet composition and VMT distribution. Refueling emissions for NEIs 2011, 2014, and 2017 are provided separately from vehicle emissions for each county, and we scale them up according to the total fleet VMT in each county. NEI emissions include only all on- and off-network processes and refueling, but not other nonroad life cycle emissions, which are therefore also not included in our study. For the state of California, we complement missing GHG emissions in NEI 2008 and missing N_2_O emissions in NEI 2014 with GHG emissions data from the California Air Resources Board (37).

### Health Impacts.

We assess mortality attributable to chronic exposure to PM_2.5_ because it accounts for the vast majority of overall monetized air pollution impacts. Our analysis does not consider the following: 1) the effects of ozone, 2) the impacts of acute exposure, or 3) the many nonfatal effects of PM_2.5_ exposure. Mortality attributable to ozone has been estimated to be about an order of magnitude smaller than mortality attributable to PM_2.5_ for the transportation sector in the United States (1, 3, 12). Mortality attributable to acute exposure to PM_2.5_ has also been found to be much smaller than mortality attributable to chronic exposure. Even in China, where the haze episodes are severe, mortality attributable to acute exposure was an order of magnitude smaller (38). Finally, the incidence of nonfatal outcomes attributable to PM_2.5_ is higher, but an analysis of the benefits of the Clean Air Act from 1990 to 2020 attributes over 90% of the estimated monetized benefits in 2020 to PM_2.5_-attributable mortality (11).

We estimate the marginal impact on mortality associated with marginal changes in emissions of each of the five pollutants in each county. We follow the method used by Choma et al. (20), refined with county-level age-specific baseline mortality data and baseline ambient PM_2.5_ levels at a finer spatial resolution (1 km) (21). We also relax the assumption made by Choma et al. (20) that impacts occur in the same place as emissions to better capture spatial variability in baseline mortality rates and ambient PM_2.5_ levels. Baseline ambient PM_2.5_ levels affect marginal impacts if, as we assume, the relationship between ambient PM_2.5_ concentrations and mortality risk is nonlinear. The approach is based on three main components: 1) an estimate of the impacts of the ground-level emissions on fine particle exposure (i.e., changes in concentrations), 2) a CRF relating PM_2.5_ concentration to mortality risk, and 3) baseline mortality rates.

For the first component, we use the ISRM (2, 22, 36), which estimates changes in fine particle concentrations in a receptor cell as a function of emissions of each of the five air pollutants covered in this study in each cell. It has the advantage of fine spatial resolution with variable cell sizes that are as small as 1 × 1 km for densely populated areas. We map ISRM cells to US counties weighting by population as described in Choma et al. (20) but using more recent population estimates. We use 5-y estimates of population at the census block group level from the 2015 to 2019 American Community Survey (39) and geography boundaries from the US Census Bureau (40, 41).

For the second component, we use the GEMM CRF (16) because it incorporates the most recent epidemiological evidence coming from studies in North America, extending the range of exposures and risk estimates to concentrations as low as 2.4 μg/m^3^. The authors (a collaboration among research groups that includes those responsible for 15 of the largest individual cohorts to date) fit a unified model to individual-level data from these cohorts as well as published data for 26 other cohorts. Burnett et al. (16) provide different sets of coefficients, and we use the age-specific GEMM NCD+LRI coefficients, encompassing all nonaccidental deaths. We use the GEMM version that includes evidence from a recent Chinese male cohort study, although this inclusion did not substantially affect GEMM’s CRF and also did not result in substantial differences when estimating mortality in the United States in Choma et al. (20). The GEMM CRF is concave in ambient PM_2.5_ concentrations; therefore, risk ratios are a function of the baseline ambient PM_2.5_ concentration. This nonlinearity is supported by two other important recent syntheses of the epidemiological evidence, both of which estimated concave CRFs: GBD IER (17, 18) and the metaregression by Vodonos et al. (32). Nonlinearities in effects with respect to baseline PM_2.5_ concentrations are particularly relevant at the very low concentrations (<10 μg/m^3^) experienced by the vast majority of the US population (21).

We calculated baseline PM_2.5_ concentrations for each county by weighting concentration estimates at a 1-km resolution (21) by population at a census block level (*n* = 11,007,989) from the 2010 Decennial Census (42). We assigned an annual average PM_2.5_ level to each census block using the distances from block centroids to the centroids of the four closest 1-km model cells, weighting by inverse distance. The estimated concentrations result in national population-weighted averages of 10.5 μg/m^3^ in 2008 and 7.5 μg/m^3^ in 2016, similar to the decrease from 10.9 to 7.7 μg/m^3^ in those years shown by the EPA based on data from 406 trend sites (10). We used model outputs from Di et al. (21) for 2016, the last year available, as a proxy for 2017. The EPA data show a small increase from 7.7 μg/m^3^ in 2016 to 8.1 μg/m^3^ in 2017.

For the third component we used, for each county and GEMM age group, nonaccidental baseline mortality data from the Centers for Disease Control and Prevention (CDC) Wonder database (23) and population data from HHS (29). For each county and age group, we calculated a mortality rate from 5 y of deaths and population counts and multiplied it by the population of the year of interest (2008 and 2017) to estimate the number of deaths. For 2008, we used data from 2006 to 2010, whereas, for 2017, we used data from 2014 to 2018, the last 5 y available. For counties with <50 death counts in any given age group in the 5-y period, we used the state-level mortality rates for that age group and applied it to the county population for that age group. In our sensitivity analysis using other CRFs that estimate risks for all-cause mortality, we used the same procedure and data sources but collected data for all ages and causes of death.

Marginal impacts for emissions of each pollutant are assessed with Eq. **1**. The ISRM provides increases in concentrations (Δ*C*) caused by an emission of 1 μg/s (0.03 kg/year); therefore, our marginal impacts are assessed for emissions of 0.03 kg of each pollutant. While GEMM’s CRF is nonlinear in ambient PM_2.5_ levels, even if we eliminated all 19,800 traffic-related PM_2.5_-attributable deaths we estimate for 2017, the change in GEMM’s slope and in the marginal effect would be smaller than 10%. This would cause an average error in our model that is smaller than 5% because we apply the same marginal impacts to all transportation emissions. Our model also does not include deaths occurring outside of the United States. We assume that any air pollution exports to other countries is very small—even exports across US states are responsible for just 24% of the total attributable deaths.

[1] $$\mathrm{Marginal}\;\mathrm{Impac}t_{s,p}=\underset{r}{\sum }\underset{a}{\sum }M_{r,a}\left(\Delta C_{s,r,p}\right)=\underset{r}{\sum }\underset{a}{\sum }\left(\frac{RR_{a,d}\left(C_{r}+\Delta C_{s,r,p}\right)-RR_{a,d}\left(C_{r}\right)\;}{RR_{a,d}\left(C_{r}\right)}\times M_{r,a}\right),$$

where *C* is the PM_2.5_ concentration [μg/m^3^], Δ*C* is the increase in concentrations [μg/m^3^], *RR* is the risk ratio from the CRF [dimensionless], and *M* is the outcome measure [mortality in deaths/year], which is cause-specific (nonaccidental) for GEMM and all-cause for the other CRFs.

The index *s* represents the sources (52,411 InMAP cells, which we map to 3,108 US counties), *r* are the receptors (52,411 InMAP cells, which we map to 3,108 US counties), *p* = 1,2,…,5 are the five pollutants, and *a* are the age groups (12 for GEMM and 1 for all-cause mortality). In our analysis of impacts occurring in state and out of state, we sum receptors accordingly (*r* inside the state of *s* and *r* outside the state of *s*).

The computational implementation of Eq. **1**, including the mapping of InMAP cells to US counties, is shown in Eq. **2**. Eq. **2** is applied to each pollutant separately, as the ISRM is specific to each pollutant. The other matrices and parameters are the same for all pollutants.

[2] $$MI=P\times \mathrm{ISRM}\times P^{T}\times M\times k\times \mathrm{VSL},$$

where ***MI*** is a 3,108 × 3,108 matrix where *MI_i,j_* is the impact [2017 US dollars] occurring in county *j* (receptor) as a consequence of 1 metric ton of emissions in county *i* (source); ***P*** is a 3,108 × 52,411 matrix where element *P_ij_* is the percentage of the population of county *i* that is within InMAP cell *j*; ***ISRM*** is a 52,411 × 52,411 matrix where *ISRM_ij_* is the increase in concentration (Δ*C*) [μg/m^3^] in InMAP cell *j* (receptor) as a consequence of emissions of 1 μg/s in InMAP cell *i* (source); ***M*** is a 3,108 × 3,108 diagonal matrix where *M_ij_* is the increase in mortality (deaths) for an increase in 1 μg/m^3^ in ambient concentration if *i* = *j* and *M_ij_* = 0 if *i* ≠ *j*; *k* = 10^12^/(24 × 3,600 × 365), representing the conversion from 1 μg/s to 1 metric ton/year; and *VSL* is the VSL [2017 US dollars] in the case of monetized damages.

***M*** was calculated as ***M*** = Dg(diag(***D*** × ***S^T^***)), in which ***D*** is a 3,108 × *N_a_* matrix, where *D_ij_* is the number of deaths in county *i* and age group *j*; ***S*** is a 3108 × *N_a_* matrix, where *S_ij_* is the percent increase in baseline mortality in county *i* and age group *j* for an increase in ambient concentrations of 1 μg/m^3^; diag(***X***) denotes the vector containing the diagonal elements of matrix ***X***; and Dg(***x***) denotes the square matrix where off-diagonal elements are 0 and diagonal elements are the elements of vector ***x***.

For GEMM, ***D*** contains data only for nonaccidental mortality, and ***S*** only applies to those causes of death. For the other CRFs, all deaths are accounted for in ***D***, and ***S*** applies to all-cause mortality. *N_a_* is the number of age groups, which equals 12 for GEMM and 1 for the other CRFs.

Matrix ***P*** maps InMAP cells to counties, allocating county death counts (in the case of receptors) and emissions (in the case of sources) to the InMAP cells within them, weighting by population and implicitly assuming that within-county spatial distributions of mortality and vehicle emissions follow that of population.

The shape of the CRF at very low concentrations is particularly uncertain, with a lack of evidence for exposures below the GEMM minimum (2.4 μg/m^3^). We assign zero marginal impacts for changes in concentrations in counties below that exposure level, which include 0.02% of the population in 2017 and 0% in 2008. Moreover, while we calculate marginal impacts for any level above 2.4 μg/m^3^, any policy that leads to meaningful public health improvements will lower concentrations by more than a marginal amount. If we consider that amount to be 0.5 μg/m^3^, it would include 0.1% of the population in 2017 and 0.006% in 2008 (i.e., that experienced baseline levels <2.9 μg/m^3^ or no more than 0.5 μg/m^3^ above the threshold). In these rare cases, while we estimate positive marginal benefits, meaningful policies of emission reductions might have little effect. We use the same minimum level of 2.4 μg/m^3^ for all CRFs, extending the risk estimates by Vodonos et al. (32) and Krewski et al. (34) to that level, even as the studies were not able to estimate risks for such low exposures.

We present our air pollution results in 2017 US attributable deaths as well as their monetized value in 2017 US dollars. The monetary value is calculated using a $10.2 million VSL, reflecting the $9.3 million in 2014 used by HHS (35) adjusted to 2017 for inflation [US gross domestic product (GDP) deflator from World Bank (43)] and income [median usual weekly earnings from US Department of Labor (44)], the latter assuming an income elasticity of the VSL of 1. There is a “cessation lag” between changes in emissions and changes in mortality, and we apply the cessation lag structure recommended by the EPA’s Advisory Council on Clean Air Compliance Analysis (45), discounting benefits using a 3% discount rate in our monetized results. In the EPA’s recommended structure, 30% of the benefits occur in the first year, another 50% uniformly in years 2 to 5, and the remaining 20% uniformly between years 6 and 20. This results in a net present value of 0.89 × $10.2 million, or $9.1 million, for each fatality. Our results presented as attributable deaths, on the other hand, are simply the undiscounted sum of attributable deaths that occur in years 1 through 20.

*SI Appendix*, Figs. S7–S10 contain distributions of marginal impacts per metric ton under different CRFs. Full datasets are provided in the *SI Appendix*.

### GHG Impacts.

We use 100-y global warming potentials (GWPs) with climate-carbon feedback (34 for CH_4_ and 298 for N_2_O) from the Fifth Assessment Report of the Intergovernmental Panel on Climate Change (46). We present monetized GHG impacts using an average regulatory SCC for 2020 with a 3% per year discount rate ($42 per metric ton in 2007 US dollars) (31). To illustrate the uncertainty in the SCC, we explore the sensitivity of our results to high-impact scenario regulatory SCC ($123 per metric ton in 2007 US dollars) (31). We adjust both values to 2017 US dollars for inflation using the US GDP deflator (43), resulting in values of $49 per metric ton and $144 per metric ton in 2017 US dollars for the average and high-impact scenarios, respectively.

### Model Implementation.

Our model was implemented in R version 3.5.1 (47) using R packages “rgeos” version 0.5-2 (48), “raster” version 2.6-7 (49), “sp” version 1.3-1 (50), “geosphere” version 1.5-10 (51), “ncdf4” version 1.16 (52), “nlme” version 3.1-137 (53), “mgcv” version 1.8-24 (54), “viridis” version 0.5.1 (55), “viridisLite” version 0.3.0 (56), and “gridExtra” version 2.3 (57).

We provide the computer code used in this manuscript at https://doi.org/10.7910/DVN/V3SXIM.

A previous version of this manuscript has been included in Ernani F. Choma’s doctoral dissertation at Harvard University (58).

## Supplementary Material

Supplementary File

Supplementary File

Supplementary File

Supplementary File

Supplementary File

Supplementary File

## Data Availability

Computer code and data have been deposited in Harvard Dataverse (https://doi.org/10.7910/DVN/V3SXIM) (59). Together with the code, we provide: the data used that is not publicly available (also provided in Dataset S5); a portion of the publicly-available data used and detailed instructions of how and where to download the remaining publicly available datasets used in this manuscript; and data files with our model results. We share all our model results, including matrices MI described in Eq. **2**. Eighty matrices MI are provided, which were estimated for each of five pollutants (primary PM_2.5_, SO_2_, NO_x_, NH_3_, and VOCs), four CRFs [GEMM (16), the two approaches by Vodonos et al. (2018) (32), and Krewski et al. (2009) (34)], two baseline ambient PM_2.5_ levels (for years 2008 and 2017), and two baseline mortality data years (2008 and 2017). We also provide a summary of supplemental results and data in Datasets S1–S4. Datasets S1–S4 contain marginal damages per metric ton of emissions; passenger LDV impacts per mile; vehicle emission factors per mile; and refueling emission factors; respectively. Previously published data were used for this work (22–30, 33, 37, 39–44). The only data not publicly available are the county-level population-weighted annual average PM_2.5_ concentrations in 2008 and 2017. These were based on the model by Di et al. (21) in our reference list, and we include them with the submission (Dataset S5). A full description of Datasets S1–S5 is found in the *SI Appendix*.

## Change History

February 25, 2022: Figure 3 and the SI Appendix have been updated; please see accompanying Correction for details.
