# Supplementary material for: Health benefits of decreases in on-road transportation emissions in the United States from 2008 to 2017
Source: Proc Natl Acad Sci U S A. 2021 Dec 13;118(51):e2107402118. doi: 10.1073/pnas.2107402118 (PMC8713776; doi:10.1073/pnas.2107402118)
Supplement: Supplementary File [file pnas.2107402118.sapp.pdf]

PNAS
www.pnas.org

Supplementary Information for
Health benefits of decreases in on-road transportation emissions in the United States from 2008 to 2017.

Ernani F. Choma* [a,b], John S. Evans[b], José A. Gómez-Ibáñez[c], Qian Di[d], Joel D. Schwartz[b], James K. Hammitt[e,f], and John D. Spengler[b].
[a] Population Health Sciences, Harvard University, Boston, MA, 02115.
[b] Department of Environmental Health, Harvard T.H. Chan School of Public Health, Boston, MA, 02115.
[c] Harvard Kennedy School of Government, Cambridge, MA, 02138.
[d] Vanke School of Public Health, Tsinghua University, Beijing, China, 100084
[e] Department of Health Policy and Management, Harvard T.H. Chan School of Public Health, Boston, MA, 02115.
[f] Toulouse School of Economics, Université Toulouse Capitole.

*Corresponding Author: Ernani F. Choma
Email: echoma@hsph.harvard.edu

This PDF file includes:

Supplementary text
SI References
Figures S1 to S10
Tables S1 to S4
Descriptions for Supplemental Datasets S1 to S5

Other supplementary materials for this manuscript include the following:

Datasets S1 to S5

Supplementary Information Text

1. Vehicle classifications

We use EPA's classification of vehicle types [1], shown in Table S1.

Vehicle types in NEI 2008 are different than in the other NEIs due to changes EPA's modeling process in 2011 (version 2) – when it moved from MOBILE to MOVES – and there is not a one-to-one correspondence [1-4]. We converted vehicle types in the 2008 NEI to the types used in later versions according to EPA's conversion rule based on overall VMT in 2011 [1, pp. 124-126]. The difference seems small for LDVs, as it is limited to commercial light duty trucks, which represent only a small portion of LDV VMT. For HDVs, on the other hand, the changes in classification were more substantial.

We also had a very small number of counties where the data was inconsistent for one or more vehicle types, with either (i) emissions > 0 and VMT = 0 (or missing); or (ii) emissions = 0 (or missing) and VMT > 0. These are only a minor part of emissions. Emissions in case (i) summed up to just $2*10^{-6}$ % of the total emissions across pollutants and years. Counties in case (ii) not exceeding 1.5% of either 2017 VMT or total emissions for any combination of pollutant, year, and vehicle type, with the exception of GHGs in California. In such cases, for each NEI year, we applied mean emission factors, with the exception of GHGs in California. If the county was outside of California, we applied U.S. means (excluding California). If the county was in California, we applied California's statewide mean emission factors. We separated California because the EPA does not model on-road emissions for the state of California – emissions are provided directly by the state [5].

GHG (i.e. CO_2, CH_4, and N_2O) emissions in 2008 and N_2O emissions in 2014 were missing for the state of California in the respective NEIs [3,6], so we complement them with data from the California Air Resources Board (CARB) [7]. GHG impacts are not location dependent, so in each case we applied state-wide emission factors for each of the four vehicle classes (motorcycles, LDVs, buses, and HDTs) using CARB's sector level 4 classification (Table S2). Although we do not adjust CARB-reported emissions of GHGs in 2008 (or N_2O in 2014), we note that CARB's CO_2-eq. emissions in subsequent years (2011 and 2014) were lower than those reported in the respective NEIs by 13% and 11%, respectively.

2. Fleet composition and VMT adjustments

EPA's data indicates a substantial decrease in light commercial truck VMT over the period, while some types of heavy-duty trucks show a substantial increase (Table S3). This could be due to differences in vehicle classifications, even though the difference occurs between NEIs 2011 and 2014, and NEI 2011 v2 (which we use) already uses the current vehicle classification. Commercial light-duty trucks make up only a small percentage of the LDV VMT, but would make up a large percentage of the HDV VMT if they were heavy-duty vehicles.

We investigated the possible effect of vehicle classification in our results by creating an alternative VMT adjustment. Impacts for the 2017 EFs scenario are not affected since they are the impacts of emissions in 2017, but impacts in counterfactual scenarios for emission factors in previous years depends on adjusting VMT to 2017 levels. Our base results apply county-level vehicle-type emission factors per mile (EFs) to 2017 VMT from [8] – essentially scaling results from previous years according to changes in VMT of each vehicle type in each county. We construct an alternative adjustment, where we scale changes in VMT for each of: motorcycles, passenger cars, and all other vehicles combined. Classification of motorcycles and cars is unlikely to have changed, and we combine all other vehicle types together in case a portion of light-duty commercial trucks were re-classified as passenger trucks or heavy-duty vehicles.

In our base case, we estimate 48,200 deaths attributable to $PM_{2.5}$ in 2017 in the 2008 EFs scenario, whereas the alternative VMT adjustment leads to 46,500, regardless of whether this alternative adjustment is done individually for each county's fleet composition or whether the same proportional adjustment is carried out based on the entire country's fleet composition change. VMT effects led to an increase in 22% since 2008 in our base case, a figure that drops to 17% in the alternative adjustment when the alternative adjustment is done at a county level, or to 18% when the alternative adjustment is done at a national level. This might seem counterintuitive since total fleet VMT increased by just 7% in the period, but a decrease of car VMT and an increase of larger vehicles leads to higher effects, even if it there is some uncertainty about whether these other vehicles are light or heavy-duty trucks. VMT for passenger cars – responsible for about a quarter of impacts in 2008 EFs prior to VMT adjustment – decreased by 15%, whereas the VMT for all other vehicles (except motorcycles) increased by 30%, and they make up about three quarters of impacts. This leads to VMT effects that are much larger than overall increases in VMT.

3. Supplementary Results

Figures S1-S11 and Table S4 show our supplementary results.

SI References:

1. U.S. Environmental Protection Agency, Population and Activity of On-Road Vehicles in MOVES 2014. (2016). https://nepis.epa.gov/Exe/ZyPDF.cgi?Dockey=P100O7PS.pdf (accessed 27 August 2020).
2. U.S. Environmental Protection Agency, 2011 National Emissions Inventory, version 2: Technical Support Document. (2015). https://www.epa.gov/sites/production/files/2015-10/documents/nei2011v2_tsd_14aug2015.pdf (accessed 27 August 2020).
3. U.S. Environmental Protection Agency, Data from “2008 National Emissions Inventory (NEI)”, 2008nei_v3. https://www.epa.gov/air-emissions-inventories/2008-national-emissions-inventory-nei-data (accessed 30 April 2021).
4. U.S. Environmental Protection Agency, Data from “2011 National Emissions Inventory (NEI)”, 2011nei_v2. https://www.epa.gov/air-emissions-inventories/2011-national-emissions-inventory-nei-data (accessed 30 April 2021).
5. U.S. Environmental Protection Agency, 2017 National Emissions Inventory: January 2021 Updated Release, Technical Support Document. (2021). https://www.epa.gov/sites/production/files/2021-02/documents/nei2017_tsd_full_jan2021.pdf (accessed 07 June 2021).
6. U.S. Environmental Protection Agency, Data from “2014 National Emissions Inventory (NEI)”, 2014nei_v2. https://www.epa.gov/air-emissions-inventories/2014-national-emissions-inventory-nei-data (accessed 30 April 2021).
7. California Air Resources Board, Data from “GHG Inventory Data Archive: 2018 Edition: Years 2000-2017.” https://ww3.arb.ca.gov/cc/inventory/pubs/reports/2000_2016/ghg_inventory_by_sector_all_00-16.xlsx (accessed 27 August 2020).
8. U.S. Environmental Protection Agency, Data from “2017 National Emissions Inventory (NEI)”, 2017nei_v1 (Apr 2020). https://www.epa.gov/air-emissions-inventories/2017-national-emissions-inventory-nei-data (accessed 30 April 2021).
9. R. T. Burnett *et al.*, An Integrated Risk Function for Estimating the Global Burden of Disease Attributable to Ambient Fine Particulate Matter Exposure. *Environ. Health Perspect.* **122**, 397-403 (2014). https://doi.org/10.1289/ehp.1307049.
10. A. Vodonos, Y. Abu Awad, J. Schwartz, The concentration-response between long-term $PM_{2.5}$ exposure and mortality; A meta-regression approach. *Environ. Res.* **166**, 677-689 (2018). https://doi.org/10.1016/j.envres.2018.06.021.
11. Interagency Working Group on Social Cost of Greenhouse Gases, Technical Support Document: – Technical Update of the Social Cost of Carbon for Regulatory Impact Analysis – Under Executive Order 12866 (2016). Interagency Working Group on Social Cost of Greenhouse Gases, United States Government. https://19january2017snapshot.epa.gov/sites/production/files/2016-12/documents/sc_co2_tsd_august_2016.pdf (accessed 22 May 2020).
12. D. Krewski *et al.*, Extended Follow-Up and Spatial Analysis of the American Cancer Society Study Linking Particulate Air Pollution and Mortality (2009). Health Effects Institute, Boston MA: HEI Research Report 140. https://www.healtheffects.org/system/files/Krewski140.pdf (accessed 22 April 2020).
13. A. Vodonos. The data associated with the publication "Vodonos, Alina, Yara Abu Awad, and Joel Schwartz. "The concentration-response between long-term PM2. 5 exposure and mortality; A meta-regression approach." Environmental research 166 (2018): 677-689.". (2019). https://github.com/AlinaVod/meta_regression_pm2.5. (accessed 09 March 2021).
14. U.S. Census Bureau, Metropolitan and micropolitan statistical area population estimates and estimated components of change: April 1, 2010 to July 1, 2019 (CBSA-EST2019-alldata). https://www2.census.gov/programs-surveys/popest/datasets/2010-2019/metro/totals/cbsa-est2019-alldata.csv. (accessed 3 May 2021).
15. U.S. Census Bureau, Population, Population Change, and Estimated Components of Population Change: April 1, 2010 to July 1, 2019 (CO-EST2019-alldata).

https://www2.census.gov/programs-surveys/popest/datasets/2010-2019/counties/totals/co-est2019-alldata.csv. (accessed 3 May 2021).
16. Q. Di *et al.*, An ensemble-based model of $PM_{2.5}$ concentration across the contiguous United States with high spatiotemporal resolution. *Environment International* **130**, 104909 (2019). https://doi.org/10.1016/j.envint.2019.104909.
17. U.S. Census Bureau, Data from "2010 TIGER/Line Shapefiles with 2010 Census block geography and the 2010 Census population and housing unit counts." https://www2.census.gov/geo/tiger/TIGER2010BLKPOPHU/ (accessed 11 September 2020).

Figures and Tables

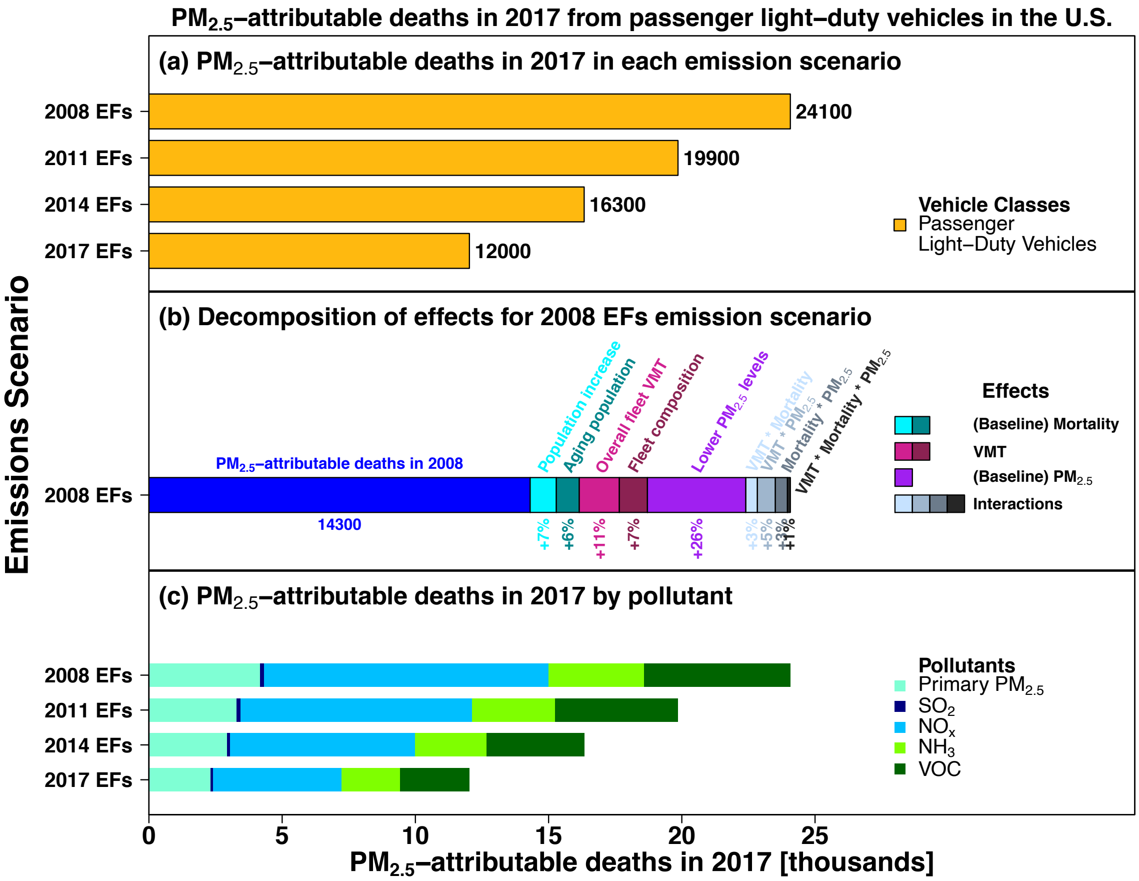

Figure S1. $PM_{2.5}$-attributable deaths caused by passenger light-duty vehicle emissions in 2017, in each of the four vehicle emissions scenarios. Panel (a) shows the total impacts. Panel (b) shows the decomposition of effects over the 2008-2017 period, for the 2008 EFs scenario. Panel (c) shows the impacts for each pollutant.

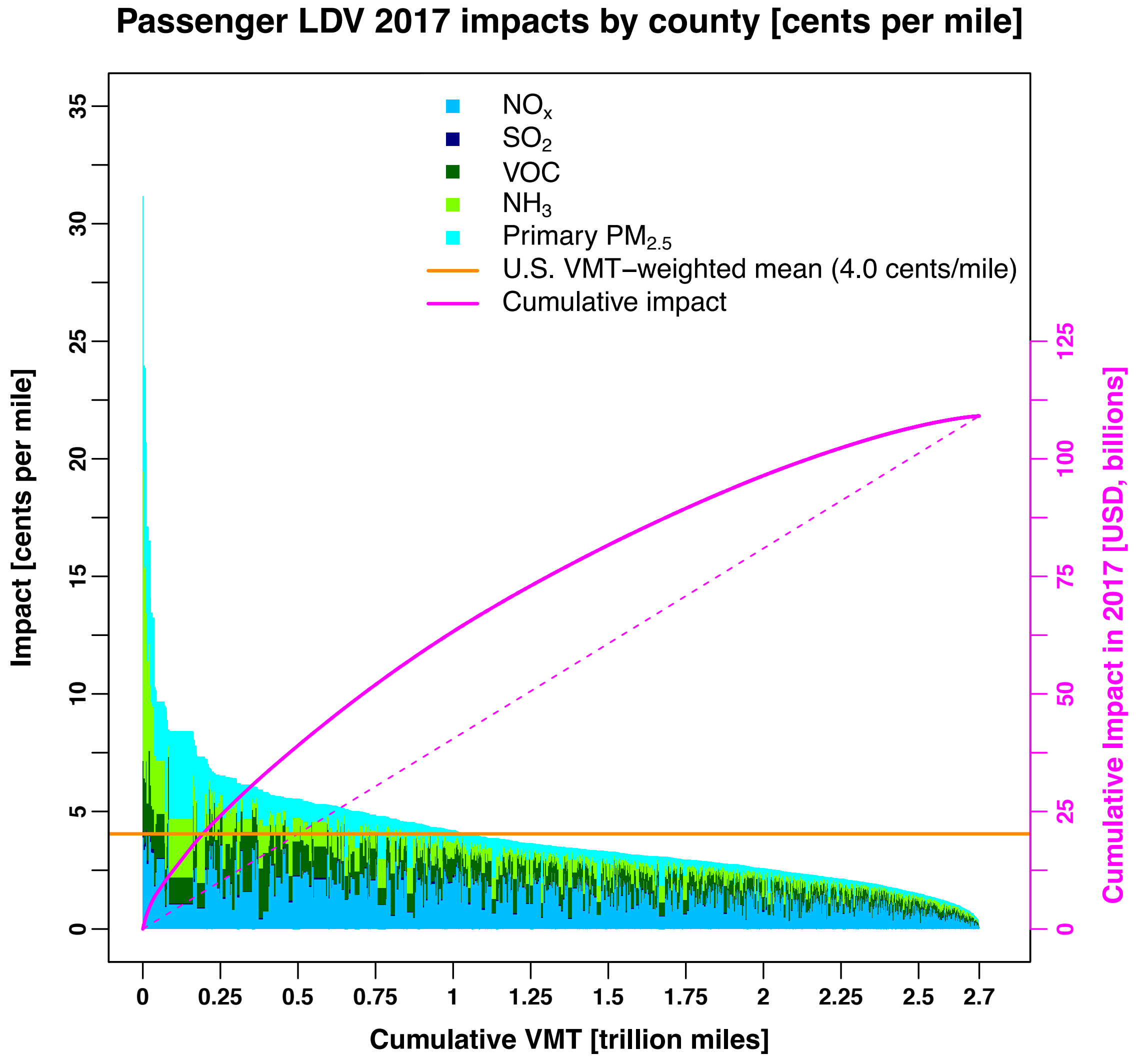

Figure S2. Impacts per mile by county in 2017, using the GEMM CRF [9] and 2017 emissions [8], ordered from highest to lowest. Areas under the curve are proportional to impacts, for each county and pollutant, and the cumulative yearly impact in 2017 is shown in magenta, in the right axis.

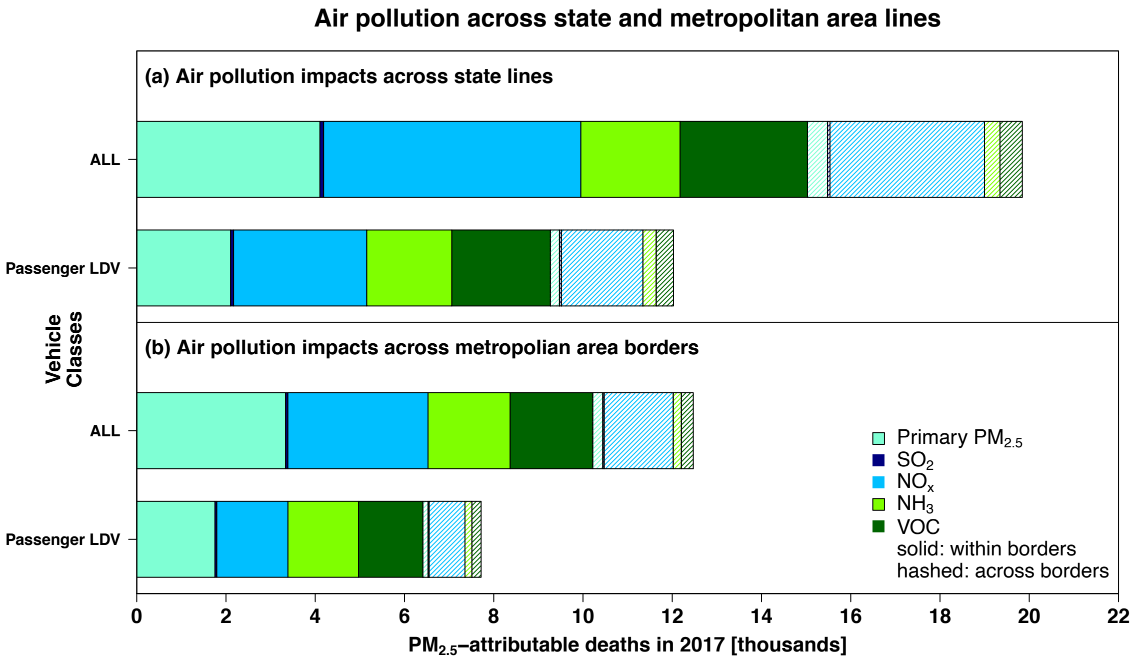

Figure S3. Deaths attributable to $PM_{2.5}$ from vehicle emissions in 2017, and the portion of which that occurs in a place that is different than where the emissions occurred. Figure S3 (a) shows the portion crossing state lines, i.e. accruing in a different state where the emissions occurred; and (b) restricts the analysis to emissions occurring in metropolitan areas, showing the portion of their impacts accruing outside the metropolitan area where emissions occurred. These are shown for both all vehicles, as well as for passenger LDVs separately.

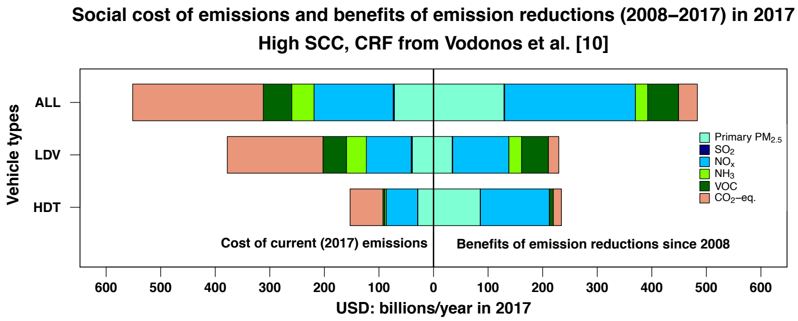

Figure S4. Social cost of emissions in 2017 (left) and benefits achieved since 2008 (right), for GHGs and each air pollutant, using the CRF from Vodonos et al.'s (2018) penalized spline approach [10] and the High Impact SCC from the Interagency Working Group on Social Cost of Greenhouse Gases [11]. If vehicles were emitting per mile as they were in 2008, benefits would not have occurred and impacts in 2017 would have been represented by the full bars (i.e. benefits shown on right side of the graph represent avoided costs; had those costs occurred, they would have been added to social costs of emissions in 2017).

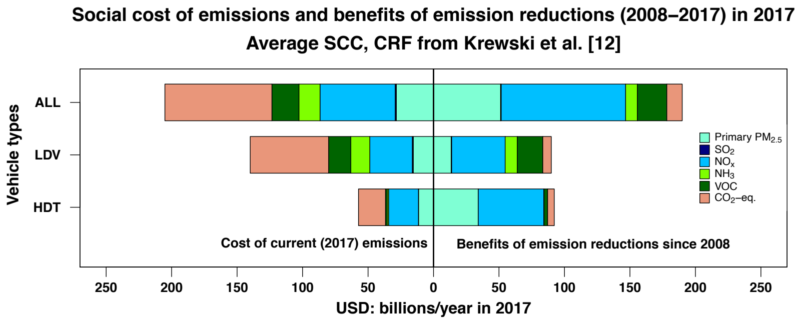

Figure S5. Social cost of emissions in 2017 (left) and benefits achieved since 2008 (right), for GHGs and each air pollutant, using the CRF from Krewski et al. (2009) [12] and the Average SCC from the Interagency Working Group on Social Cost of Greenhouse Gases [11]. If vehicles were emitting per mile as they were in 2008, benefits would not have occurred and impacts in 2017 would have been represented by the full bars (i.e. benefits shown on right side of the graph represent avoided costs; had those costs occurred, they would have been added to social costs of emissions in 2017).

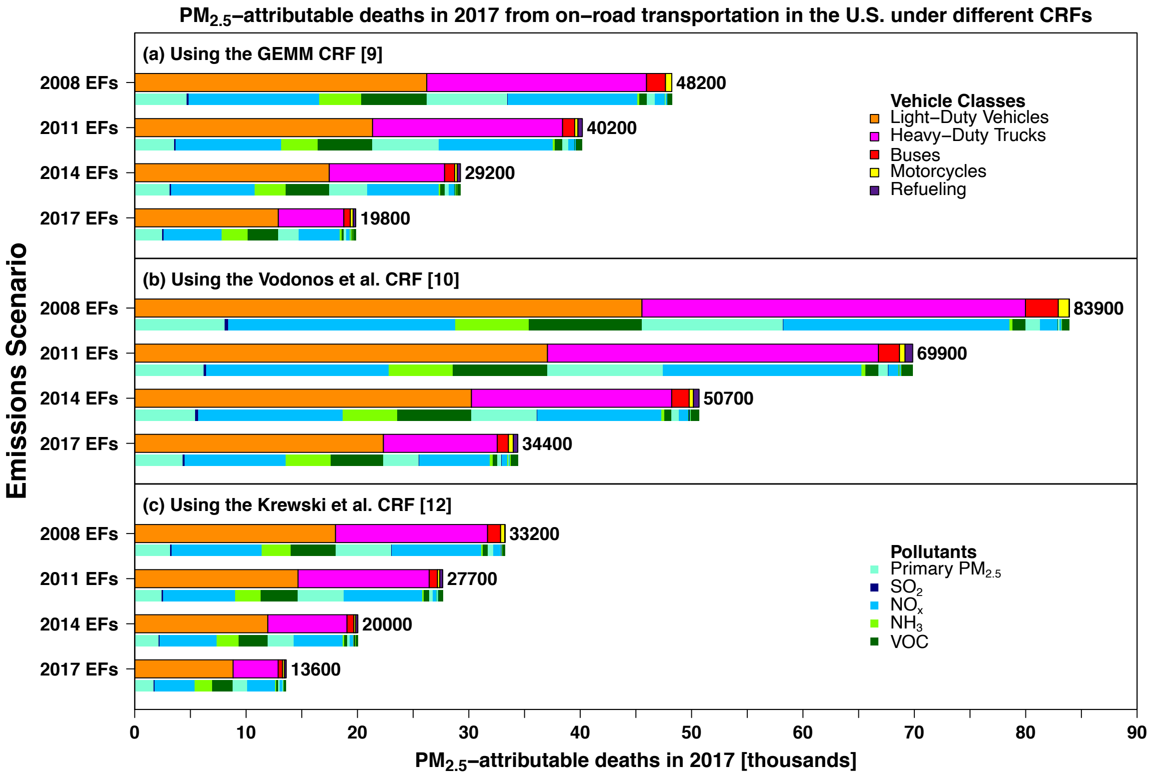

Figure S6. Deaths attributable to $PM_{2.5}$, in each of the four emissions scenarios, by vehicle class and pollutant, for each of the three CRFs considered. Panel (a) shows results using GEMM [9]. Panel (b) shows results using the CRF from Vodonos et al.'s (2018) penalized spline approach [10]. Panel (c) shows results using the CRF from Krewski et al. (2009) [12].

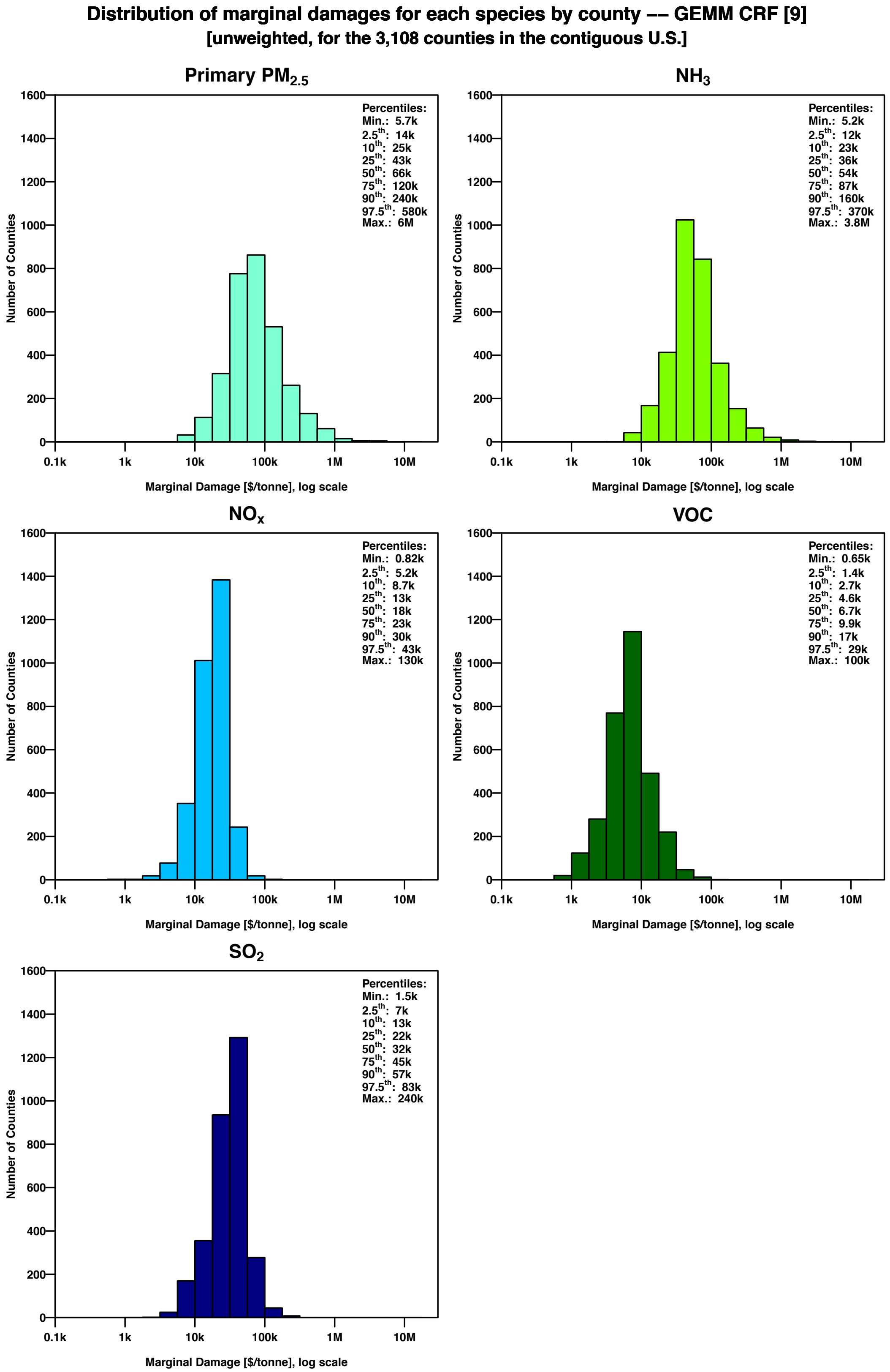

Figure S7. Distributions of marginal impacts per metric ton of emissions of each species occurring in each county in 2017, using the CRF from GEMM [9].

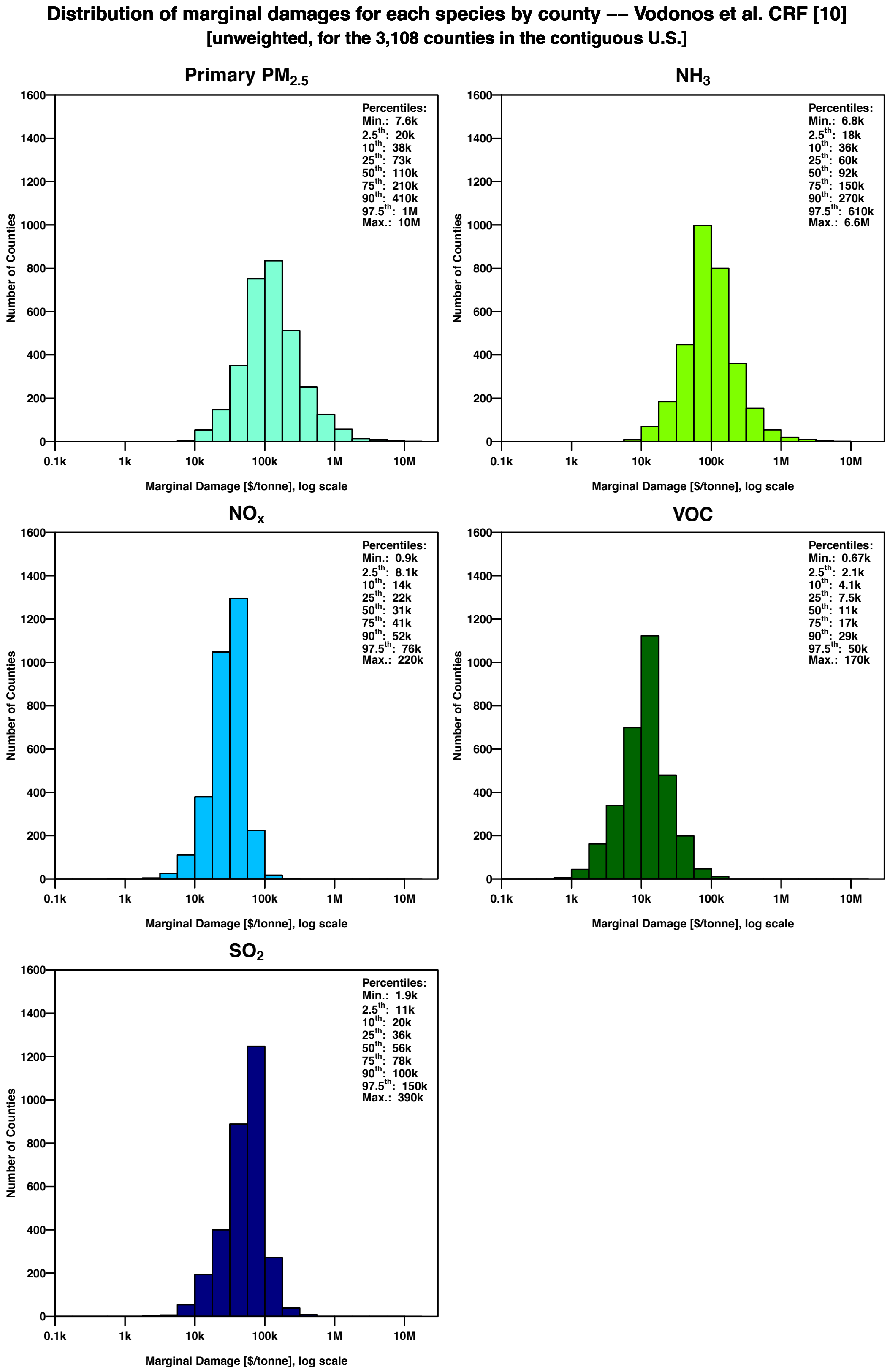

Figure S8. Distributions of marginal impacts per metric ton of emissions of each species occurring in each county in 2017, using the CRF from Vodonos et al.'s (2018) penalized spline approach [10].

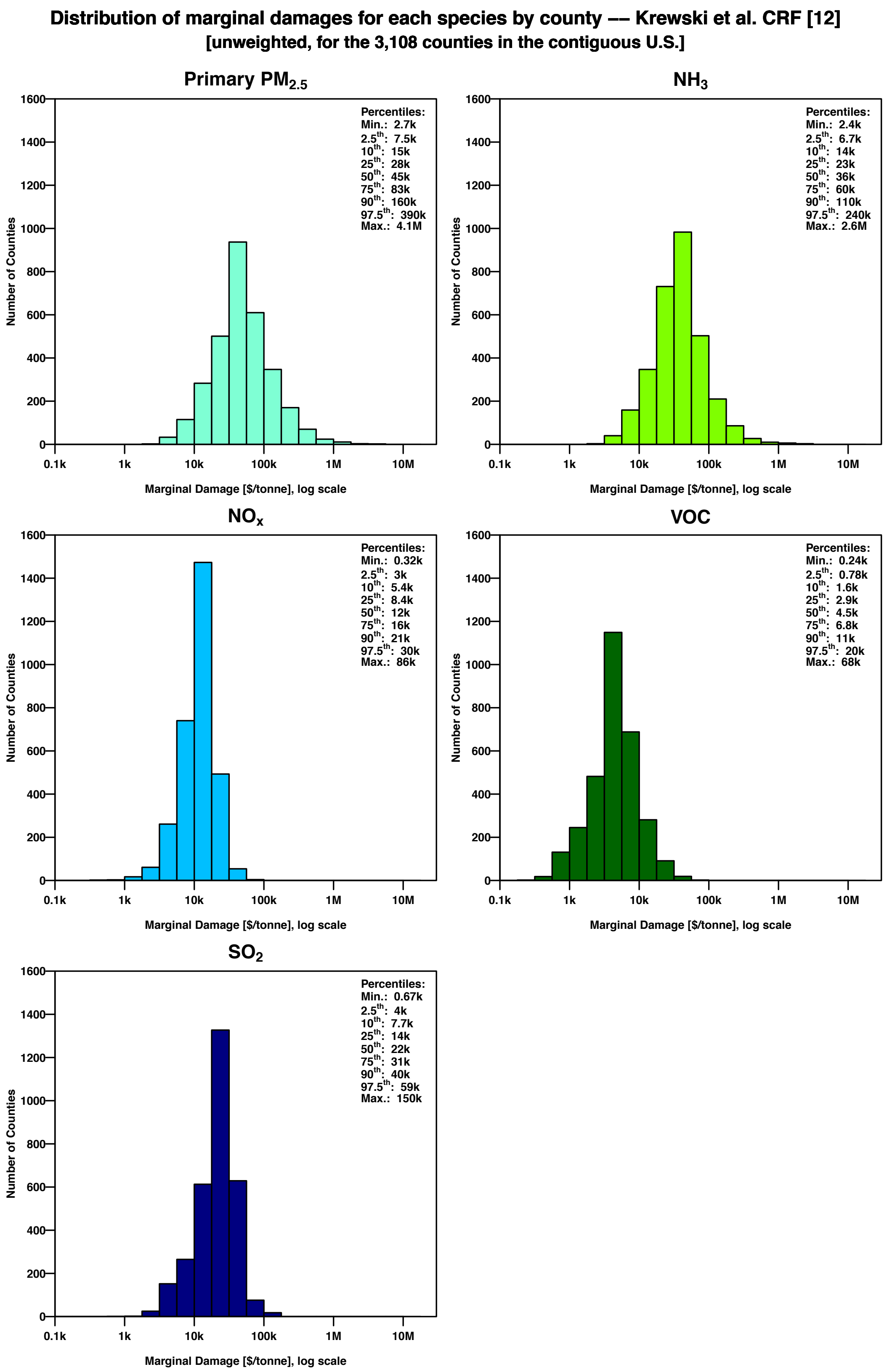

Figure S9. Distributions of marginal impacts per metric ton of emissions of each species occurring in each county in 2017, using the CRF from Krewski et al. (2009) [12].

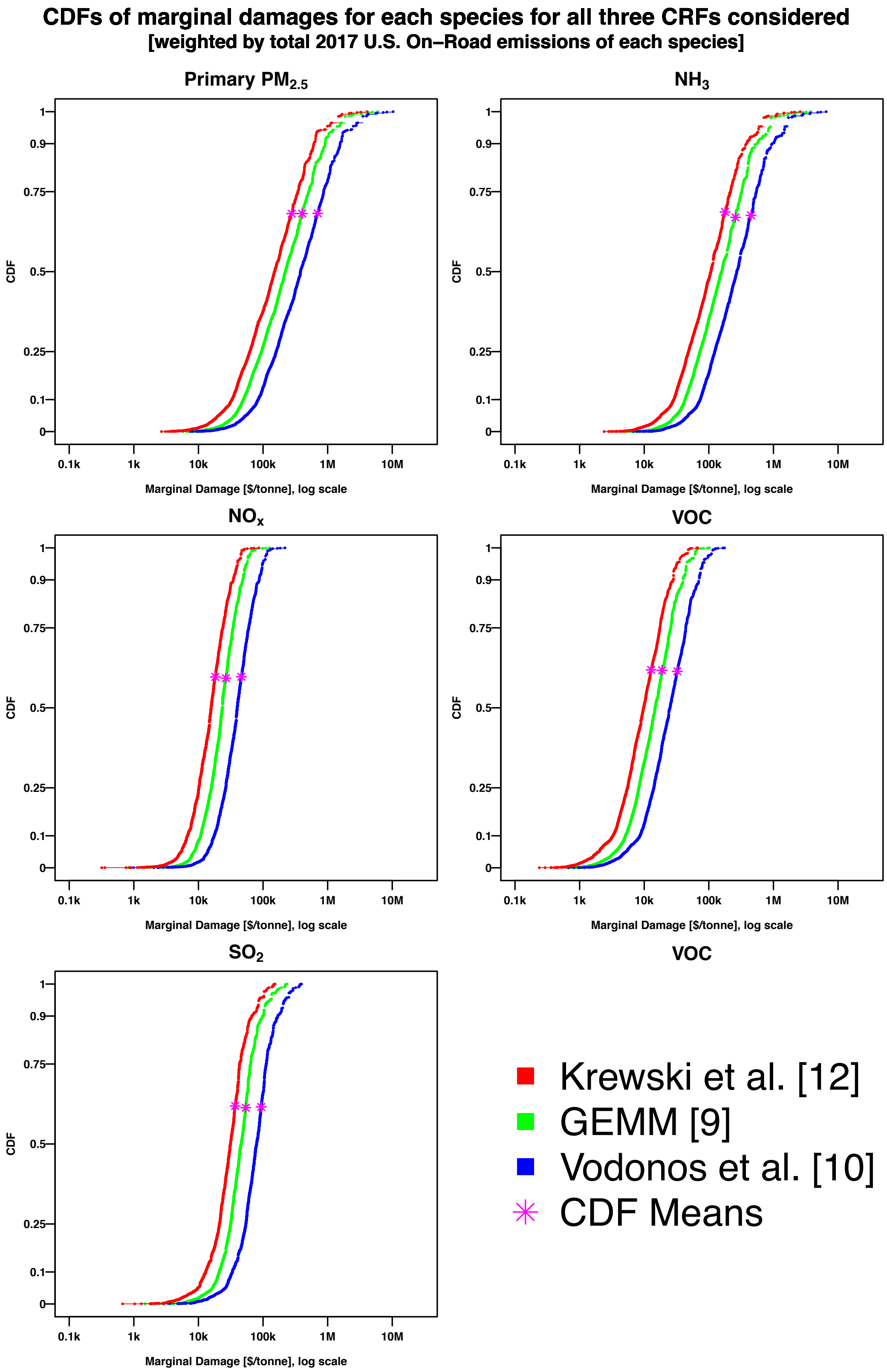

Figure S10. Cumulative distributions of marginal impacts per metric ton of emissions of each species occurring in each county in 2017, weighted by total On-Road emissions in 2017, using each of the three CRFs considered [9,10,12].

Table S1. Vehicle types and vehicle classes

EPA's vehicle type[a] (our vehicle type[b])	EPA's source type[a]	Our vehicle class[c]
Motorcycle	11	Motorcycles[d]
Passenger Car	21	LDV
Passenger Truck	31	
Light Commercial Truck	32	
Intercity Bus	41	Bus (HDV[e])
Transit Bus	42	
School Bus	43	
Refuse Truck	51	HD Truck (HDV[e])
Single Unit Short-Haul Truck	52	
Single Unit Long-Haul Truck	53	
Motor Home	54	
Combination Short-Haul Truck	61	
Combination Long-Haul Truck	62	

[a] As used in NEIs 2014 and 2017. We converted EPA's old classification, used in NEI 2008, according to the proportion of VMT of each vehicle type in 2011 [1, pp. 124-126]. For the NEI 2011, we used version 2, the most recent, which was already updated with the same MOVES 2014 vehicle types used in 2014 and 2017 [2].

[b] We use this division of vehicle types when we refer to vehicle types, such as in our VMT adjustment

[c] We do not include motorcycles as part of LDVs.

[d] Some results are presented separately for buses and HD trucks; however, when we refer to HDVs, we include both buses and HD trucks.

[e] HDV: Buses and HD Trucks

Table S2. CARB's vehicle classification

CARB's Sector Level 4 classification[a]	Our vehicle class
Motorcycle	Motorcycle
Passenger Cars	LDV
Light-duty Trucks & SUVs	LDV
Buses	Bus/HDV
Motorhomes	HD truck/HDV
Heavy-duty Trucks	HD truck/HDV

[a] Source: California Air Resources Board [7]

Table S3. VMT by vehicle type [billions of miles travelled]

Vehicle Type	2008[a]	2011[b]	2014[c]	2017[d]
Motorcycle	17	20	18	19
Passenger Car	1514	1351	1317	1281
Passenger Truck	926	1044	1268	1415
Light Commercial Truck	307	323	143	146
Intercity Bus	4	3	5	5
Transit Bus	1	3	3	3
School Bus	4	5	7	7
Refuse Truck	1	2	2	3
Single Unit Short-Haul Truck	58	55	58	72
Single Unit Long-Haul Truck	8	8	39	31
Motor Home	3	4	3	3
Combination Short-Haul Truck	50	65	54	56
Combination Long-Haul Truck	75	67	98	122
All Vehicles	2968	2949	3015	3163

[a] Source: Data from NEI 2008 [3]
[b] Source: Data from NEI 2011 [4]
[c] Source: Data from NEI 2014 [6]
[d] Source: Data from NEI 2017 [8]

Table S4. Passenger LDV impacts and emissions for each pollutant by urbanization.

	Place of emission	NO_x	VOC	Primary $PM_{2.5}$	NH_3	SO_2	CO_2-eq.
**Impacts[a] [cents/mi]**	Outside Large MSAs	1.7 (1.1 - 2.8)	0.65 (0.43 - 1.1)	0.30 (0.20 - 0.51)	0.32 (0.21 - 0.54)	0.029 (0.019 - 0.049)	2.1 - 6.2
	Large MSAs	1.6 (1.1 - 2.8)	1.1 0.75 - 1.9)	1.2 (0.86 - 2.2)	1.1 (0.80 – 2.0)	0.044 (0.031 - 0.077)	2.1 - 6.1
	10 Largest MSAs	1.6 (1.1 - 2.8)	1.1 0.82 - 2.0)	1.7 (1.2 - 3.0)	1.7 (1.2 - 3.0)	0.065 (0.045 - 0.11)	2.1 - 6.1
	Contiguous U.S.	1.6 (1.1 - 2.8)	0.87 (0.59 - 1.5)	0.78 (0.54 - 1.4)	0.74 (0.51 - 1.3)	0.037 (0.025 - 0.063)	2.1 - 6.1
**Emission Factor[b] [g/mi]**	Outside Large MSAs	0.78	0.56	0.018	0.03	0.007	431
	Large MSAs	0.47	0.37	0.016	0.027	0.0063	422
	10 Largest MSAs	0.40	0.32	0.015	0.026	0.0069	425
	Contiguous U.S.	0.62	0.46	0.017	0.029	0.0066	426
**Marginal cost per metric ton[c] [10^3 USD]**	Outside Large MSAs	21 (14 - 36)	12 (7.7 - 20)	160 (110 - 280)	110 (70 - 180)	41 (28 - 71)	0.049 - 0.144
	Large MSAs	34 (23 - 59)	29 (20 - 51)	800 (550 - 1400)	430 (300 - 750)	70 (48 - 120)	0.049 - 0.144
	10 Largest MSAs	40 (29 - 71)	36 (25 - 63)	1100 (810 - 2000)	640 (450 - 1100)	94 (66 - 160)	0.049 - 0.144
	Contiguous U.S.	26 (18 - 45)	19 (13 - 32)	460 (310 - 790)	260 (180 - 450)	55 (38 - 95)	0.049 - 0.144

Values between parenthesis for the air pollutants reflect the uncertainty in the C-R, with the lower bound calculated using Krewski et al. (2009) [12] and the upper bound calculated using the penalized spline approach from Vodonos et al. (2018) [10]. Ranges for CO_2-eq. emissions reflect the uncertainty in the SCC.

[a] VMT-weighted mean (sum of all impacts divided by sum of all VMT)

[b] VMT-weighted mean (sum of all emissions divided by sum of all VMT)

[c] Emissions-weighted mean (sum of all impacts divided by sum of all emissions)

Supplementary dataset descriptions (separate files)

Dataset S1 (separate file). Supplementary results: Marginal damages per metric ton of emissions.

This dataset contains 107 columns x 3,108 rows. Each row represents a county. The columns 8-107 are marginal damages for 1 metric ton of ground-level emissions occurring in each county, for different species, baseline inputs, and concentration-response functions.

The columns are:
Columns 1-7:
1) FIPS_STCOU: FIPS State + County Code
2) COUNTY_NAME: County Name
3) FIPS_STATE: FIPS State Code
4) STATE_NAME: State Name
5) COUNTY_IN_LARGE_MSA: Indicates (YES/NO) whether county belongs to a large Metropolitan Area (with population > 1 Million in 2017)
6) CBSA_CODE_Metro_Area_Code: CBSA Code for the metro area, if county belongs to one
7) CBSA_NAME_Metro_Area_NAME: Name for the metro area, if county belongs to one

Source for data in columns 1-7: U.S. Census Bureau [14,15]

Columns 8-107:
Columns 8-107 are named:
[Pollutant]_[Base Mortality Year]Mortality_[Baseline $PM_{2.5}$ Year]PM_[Outcome]_[CRF]

Where:
[Pollutant] is one of five species: PM25 (Primary $PM_{2.5}$); SO2; NOX; NH3; or VOC.
[Base Mortality Year] is either 2008 or 2017, indicating whether baseline mortality counts for 2008 or 2017 were used in the calculations.
[Baseline $PM_{2.5}$ Year] is either 2008 or 2017, indicating whether baseline ambient $PM_{2.5}$ concentrations for 2008 or 2017 were used in the calculations.
[Outcome] is either USD or Deaths:
USD gives the damages in 2017 USD per metric ton of emissions. It is only available for 2017 Mortality and Baseline $PM_{2.5}$ since we apply a VSL for 2017. We apply a cessation lag and discount impacts by 3%/year. We use a VSL of $10.18M, with a present value of $9.06M in 2017 after applying the cessation lag and the discount rate.
Deaths gives the damages in 2017 attributable deaths per metric ton of emissions. If multiplied by 9.06 million it will give the USD value.
[CRF] is one of the four CRFs used:
GEMM (Burnett et al., 2018);
VodonosParametric (the parametric version of Vodonos et al., 2018 [10]);
VodonosSpline (the penalized spline version of Vodonos et al., 2018 [10]); or
Krewski (Krewski et al., 2009 [12])

Dataset S2 (separate file). Supplementary results: passenger light-duty vehicle impacts per mile.

This dataset contains 31 columns x 3,108 rows. Each row represents a county. The columns 8-31 are marginal damages for 1 mile driven by passenger light-duty vehicles (passenger cars or passenger light-duty trucks) in each county, in cents per mile. These are provided for 2017, for each species and concentration-response functions. All use 2017 mortality rates, baseline ambient $PM_{2.5}$ levels for 2017, $10.18M VSL with the cessation lag and 3% discount rate ($9.06M in present value in 2017).

The columns are named as:
Columns 1-7:
1) FIPS_STCOU: FIPS State + County Code
2) COUNTY_NAME: County Name
3) FIPS_STATE: FIPS State Code
4) STATE_NAME: State Name
5) COUNTY_IN_LARGE_MSA: Indicates (YES/NO) whether county belongs to a large Metropolitan Area (with population > 1 Million in 2017)
6) CBSA_CODE_Metro_Area_Code: CBSA Code for the metro area, if county belongs to one
7) CBSA_NAME_Metro_Area_NAME: Name for the metro area, if county belongs to one

Source for data in columns 1-7: U.S. Census Bureau [14,15]

Columns 8-31:
Columns 8-31 are named:
[Pollutant]_[CRF]

Where:
[Pollutant] is one of five species or sum: PM25 (Primary $PM_{2.5}$); SO2; NOX; NH3; VOC; or SUM
Sum gives the total damages of all species per 1 mile driven.
[CRF] is one of the four CRFs used:
GEMM (Burnett et al., 2018);
VodonosParametric (the parametric version of Vodonos et al., 2018 [10,13]);
VodonosSpline (the penalized spline version of Vodonos et al., 2018 [10,13]); or
Krewski (Krewski et al., 2009 [12])

Dataset S3 (separate file). Supplementary dataset: vehicle emission factors per mile.

This dataset contains 18 columns x 161,616 rows. This dataset contains 52 rows for each of the 3,108 counties. The 52 rows represent 13 vehicle types for each of 4 National Emission Inventories (2008, 2011, 2014, and 2017).
Data sources: 2008, 2011, 2014, and 2017 National Emissions Inventories (NEI) from the U.S. Environmental Protection Agency [3,4,6,8].

The columns are:
Columns 1-7:
1) FIPS_STCOU: FIPS State + County Code
2) COUNTY_NAME: County Name
3) FIPS_STATE: FIPS State Code
4) STATE_NAME: State Name
5) COUNTY_IN_LARGE_MSA: Indicates (YES/NO) whether county belongs to a large Metropolitan Area (with population > 1 Million in 2017)
6) CBSA_CODE_Metro_Area_Code: CBSA Code for the metro area, if county belongs to one
7) CBSA_NAME_Metro_Area_NAME: Name for the metro area, if county belongs to one

Source for data in columns 1-7: U.S. Census Bureau [14,15]

Columns 8-10:
8) NEI Year, given by variable 'NEI_Data_Year'
9) The vehicle type, given by variable 'Vehicle_Type_VTYPE'
10) VMT [in miles]

Columns 11-18:
Columns 11-18 give the emission factors per mile (EF) for each pollutant, in grams/mile, and are named:
[Pollutant]_EF_grams_per_mile.
Where Pollutant is one of 8: PM25 (Primary $PM_{2.5}$); SO2; NOX; NH3; VOC; CO2; CH4; or N2O

Dataset S4 (separate file). Supplementary dataset: refueling emission factors

This dataset contains 12 columns x 9,324 rows. This dataset contains 3 rows for each of the 3,108 counties. The 3 rows represent each of 3 National Emission Inventories (2011, 2014, and 2017). NEI 2008 does not present refueling emissions separately. All emissions in this dataset are VOC emissions
Data source: 2011, 2014, and 2017 National Emissions Inventories (NEI) from the U.S. Environmental Protection Agency [4,6,8].

The columns are:
Columns 1-7:
1) FIPS_STCOU: FIPS State + County Code
2) COUNTY_NAME: County Name
3) FIPS_STATE: FIPS State Code
4) STATE_NAME: State Name
5) COUNTY_IN_LARGE_MSA: Indicates (YES/NO) whether county belongs to a large Metropolitan Area (with population > 1 Million in 2017)
6) CBSA_CODE_Metro_Area_Code: CBSA Code for the metro area, if county belongs to one
7) CBSA_NAME_Metro_Area_NAME: Name for the metro area, if county belongs to one

Source for data in columns 1-7: U.S. Census Bureau [14,15]

Columns 8-12:
8) NEI Year, given by variable 'NEI_Data_Year'
9) The vehicle type, given by variable 'Vehicle_Type_VTYPE' — we use a VTYPE of 0 for refueling
10) The sum of VMT from all vehicle types in the county [in miles], given by variable 'VMT_SUM_FROM_ALL_VEHICLE_TYPES'
11) The total NEI refueling VOC emissions for each county [in short tons, where 1 short ton = 2,000 lb.], given by variable 'VOC_EMISSIONS_SHORT_TONS'
12) The VOC emission factors per mile (EF) [g/mile, for the overall VMT from all vehicle types], given by variable 'VOC_EF_grams_per_mile'

Dataset S5 (separate file). Supplementary dataset: baseline ambient $PM_{2.5}$ concentrations

This dataset contains 9 columns x 3,108 rows. Each row represents a county.
Data source: these ambient levels were generated using the 1-km resolution estimates by Di et al. [16]. Di et al. [16] include estimates only until 2016, so we used that year (2016) as a proxy for 2017 concentrations. We produced county level estimates weighting by population at a Census block level using data from the 2010 Decennial Census [17]. We assigned a concentration to each Census Block by using the weighted average of the nearest 4 cell centroids from Di et al. [16] and weighting by inverse distance. We then aggregated the Census Blocks to counties.

The columns are:
Columns 1-7:
1) FIPS_STCOU: FIPS State + County Code
2) COUNTY_NAME: County Name
3) FIPS_STATE: FIPS State Code
4) STATE_NAME: State Name
5) COUNTY_IN_LARGE_MSA: Indicates (YES/NO) whether county belongs to a large Metropolitan Area (with population > 1 Million in 2017)
6) CBSA_CODE_Metro_Area_Code: CBSA Code for the metro area, if county belongs to one
7) CBSA_NAME_Metro_Area_NAME: Name for the metro area, if county belongs to one

Source for data in columns 1-7: U.S. Census Bureau [14,15]

Columns 8-9
8) Estimate of baseline ambient $PM_{2.5}$ levels in 2008 (county annual average) [ug/m3], given by variable ‘Ambient_PM25_2008’
9) Estimate of baseline ambient $PM_{2.5}$ levels in 2017 (county annual average) [ug/m3], given by variable ‘Ambient_PM25_2017’
